# Isolation of Novel Sesquiterpeniods and Anti-neuroinflammatory Metabolites from *Nardostachys jatamansi*

**DOI:** 10.3390/molecules23092367

**Published:** 2018-09-17

**Authors:** Chi-Su Yoon, Dong-Cheol Kim, Jin-Soo Park, Kwan-Woo Kim, Youn-Chul Kim, Hyuncheol Oh

**Affiliations:** 1 College of Pharmacy, Wonkwang University, Iksan 54538, Korea; ycs1991@naver.com (C.-S.Y.); kimman07@hanmail.net (D.-C.K.); js9181515@naver.com (J.-S.P.); swamp1@naver.com (K.-W.K.); yckim@wku.ac.kr (Y.-C.K.); 2 Hanbang Cardio-Renal Syndrome Research Center, Wonkwang University, Iksan 54538, Korea

**Keywords:** *Nardostachys jatamansi*, sesquiterpenoids, BV2 microglial cells, anti-neuroinflammation, NF-κB signaling pathway

## Abstract

*Nardostachys jatamansi* contains various types of sesquiterpenoids that may play an important role in the potency of plant’s anti-inflammatory effects, depending on their structure. In this study, five new sesquiterpenoids, namely kanshone L (**1**), kanshone M (**2**), 7-methoxydesoxo-narchinol (**3**), kanshone N (**4**), and nardosdaucanol (**5**), were isolated along with four known terpenoids (kanshone D (**6**), nardosinanone G (**7**), narchinol A (**8**), and nardoaristolone B (**9**)) from the rhizomes and roots of *Nardostachys jatamansi*. Their structures were determined by analyzing 1D and 2D NMR and MS data. Among the nine sesquiterpenoids, compounds **3**, **4**, and **8** were shown to possess dose-dependent inhibitory effects against lipopolysaccharide (LPS)-stimulated nitric oxide (NO) production in BV2 microglial cells. Furthermore, compounds **3**, **4**, and **8** exhibited anti-neuroinflammatory effects by inhibiting the production of pro-inflammatory mediators, including prostaglandin E_2_ (PGE_2_), inducible nitric oxide synthase (iNOS), and cyclooxygenase-2 (COX-2) proteins, as well as pro-inflammatory cytokines, such as interleukin (IL)-1β, IL-12 and tumor necrosis factor-α (TNF-α), in LPS-stimulated BV2 microglial cells. Moreover, these compounds were shown to inhibit the activation of the NF-κB signaling pathway in LPS-stimulated BV2 microglial cells by suppressing the phosphorylation of IκB-α and blocking NF-κB translocation. In conclusion, five new and four known sesquiterpenoids were isolated from *Nardostachys jatamansi*, and compounds **3**, **4**, and **8** exhibited anti-neuroinflammatory effects in LPS-stimulated BV2 microglial cells through inhibiting of NF-κB signaling pathway.

## 1. Introduction

The rhizomes and roots of *Nardostachys jatamansi* DC (Valerianaceae), a plant indigenous to China, India, and Tibet, have traditionally been used in the treatment of mental disorders, hyperlipidemia, hypertension, and convulsions [1]. Recently, several biological effects of the extracts from this species were reported. For instance, the ethanol extracts were shown to protect against β- amyloid-induced toxicity in SH-SY5Y cells [2]. The aqueous extracts also exhibited protective effects against 2K1C-induced cardiac hypertrophy in a rat model [3]. Furthermore, several sesquiterpenoids isolated from this species were identified as regulators of the serotonin transport system [4]. Plants belonging to the genus *Nardostachys* contain various potentially bioactive chemical components, such as monoterpenoids, sesquiterpenoids, triterpenoids, and lignans [4,5,6]. For example, we recently showed that these species contain various terpenoids with anti-neuroinflammatory effects [5].

Microglia are resident macrophages in the central nervous system (CNS), which are vital components of the innate immune system that act as the frontline of defense against foreign substances and are involved in the pro-inflammatory response [7]. Microglia express extensive pattern recognition receptors in the toll-like receptor (TLR) family to monitor for brain damage and microbial invasion of the CNS [8]. Lipopolysaccharides (LPSs) are bacterial cell wall endotoxins that are also ligands for toll-like receptor 4 (TLR4), and are thus some of the strongest stimuli of microglial activation. Binding to LPSs activate microglia, leading to the production of inflammatory cytokines and a number of neurotoxic factors that cause neuronal cell death [9,10]. Additionally, LPSs induce the production of pro-inflammatory mediators, such as inducible nitric oxide synthase (iNOS), nitric oxide (NO), prostaglandin E_2_ (PGE_2_), cyclooxygenase-2 (COX-2), interleukin (IL)-1β, IL-12, and tumor necrosis factor (TNF)-α, which can lead to neurodegenerative diseases like Parkinson’s disease (PD), Alzheimer’s disease (AD), cerebral ischemia, multiple sclerosis, and stroke [11,12,13,14,15,16,17,18].

Microglial activation is thought to be modulated by multiple signaling pathways, including the nuclear factor-kappa B (NF-κB) and mitogen-activated protein kinases (MAPKs) pathways [19]. The NF-κB pathway is known to play a vital role in the modulation of immune and inflammatory responses. Under normal conditions, NF-κB exists in the cytoplasm as p65/p50 dimers complexed with the NF-κB inhibitor kappa B, forming the NF-κB-IκB complex. However, the stimulation of microglia induces the phosphorylation of IκB proteins and the translocation of the NF-κB dimer p65/p50 into the nucleus. In the nucleus, the NF-κB dimer binds to its DNA binding site, the κB site, leading to the transcription of a number of genes that includes those coding for adhesion molecules, chemokine-inducible enzymes, and pro-inflammatory mediators like interferon-gamma (IFN-γ), NO, TNF-α, and PGE_2_ [20]. These inflammatory mediators are known to cause inflammatory reactions and neurodegenerative diseases. Therefore, suppression of the NF-κB pathway is a widely used strategy for inhibiting neuroinflammation [21,22].

## 2. Results and Discussion

### 2.1. Structure Determination of Sesquiterpenes ***1**–**9***

In our continuing study of the chemical components of *N. jatamansi*, four new nardosinone-type sesquiterpenoids (compounds **1**–**4**) and a new daucane-type sesquiterpenoid (**5**) were isolated from methanol extracts of this plant through the use of various chromatographic methods, including solvent partitioning, column chromatography, and HPLC. In addition, four known metabolites classified as nardosinone-type (**6**‒**8**) and aristolene-type sesquiterpenoids (**9**) were isolated.

Kanshone L (**1**) was isolated as a yellowish oil. Based on the analysis of ^1^H- and ^13^C-NMR data (Table 1) along with HRESIMS data, its molecular formula was determined to be C_15_H_20_O_4_, with six unsaturations. The ^1^H-NMR data (pyridine-*d_5_*) suggested the presence of two olefinic protons (δ 7.22 (H-1) and 6.03 (H-8)), two methine protons (δ 3.67 (H-4) and 3.07 (H-6))), and four methyl protons (δ 1.57 (H-12), 1.55 (H-13), 1.33 (H-14), and 1.14 (H-15)). The ^13^C-NMR and DEPT data indicated the presence of two carbonyl carbons (δ 198.9 (C-2 and -7)), two olefinic carbons (δ 125.5 (C-1) and 107.0 (C-8)), two sp^2^ quaternary carbons (δ 168.1 (C-9) and 158.6 (C-10)), two sp^3^ quaternary carbons (δ 72.2 (C-11) and 42.8 (C-5)), two sp^3^ methine carbons (δ 66.8 (C-6) and 33.9 (C-4)), a methylene carbon (δ 42.5 (C-3)), and four methyl carbons (δ 33.5 (C-12), 28.0 (C-13), 21.8 (C-14), and 16.7 (C-15)). This information accounts for four degrees of unsaturation, and thus to satisfy the required molecular formula compound **1** must be a bicyclic sesquiterpenoid with two hydroxy groups. One-bond carbon-proton correlations were determined by conducting HMQC analysis. In turn, COSY correlations from H-3 to H_3_-15 indicated the presence of a spin system corresponding to C3-C4-C15. The methyl carbon (C-15) was identified to be connected to C-4 based on the COSY spin system and the HMBCs of H-3 with C-15, H-4 with C-15, and H_3_-15 with C-3, C-4, and C-5. The correlations found with HMBC of H_3_-14 with C-4, C-5, C-6, and C-10 indicated that C-5 is a sp^3^ quaternary carbon connected to C-4, C-6, C-10, and C-14. In addition, HMBCs of H-3 with C-5, H-4 with C-3 and C-5, and H-6 with C-5 indicated the connection of C3-C4-C5-C6. C-7 was suggested to be a carbonyl carbon based on its chemical shift, and this was shown to be connected to C-6 and C-8 by the HMBCs of H-6 with C-7 and C-8 and of H-8 with C-6 and C-7. C-9 was suggested to bear a hydroxy group based on its chemical shift, and the HMBC of H-8 with C-9 and C-10 and of H-1 with C-9 and C-10 indicated that C-9 was connected to C-8, and that the sp^2^ quaternary carbon C-10 was connected to C-1, C-5, and C-9. A carbonyl carbon, C-2, was determined to be positioned between C-1 and C-3 based on the HMBC of H_2_-3 with C-1 and C-2 and of H-1 with C-3, establishing the naphthalenone ring moiety of compound **1**. The presence of an isopropyl alcohol group was confirmed based on the consideration of the chemical shift of a quaternary carbon for C-11, and the HMBCs of H_3_-12 with C-11 and C-13 and of H_3_-13 with C-11 and C-12. This isopropyl alcohol unit was then identified to be connected to C-6 based on the HMBCs of H-6 with C-11, C-12, and C-13. It appeared that kanshone L (**1**) was structurally similar to the 7-oxonardosinoperoxide previously isolated from *N. chinensis* [4]. In the ^13^C-NMR spectrum of kanshone L, a signal corresponding to the methylene carbon (C-2) observed in 7-oxonardosinoperoxide was changed to that of a carbonyl carbon. In addition, a hydroxy group was found to be located at the C-9 position in kanshone L instead of the peroxide group previously found at this position in 7-oxonardosinoperoxide.

**Figure 1:**
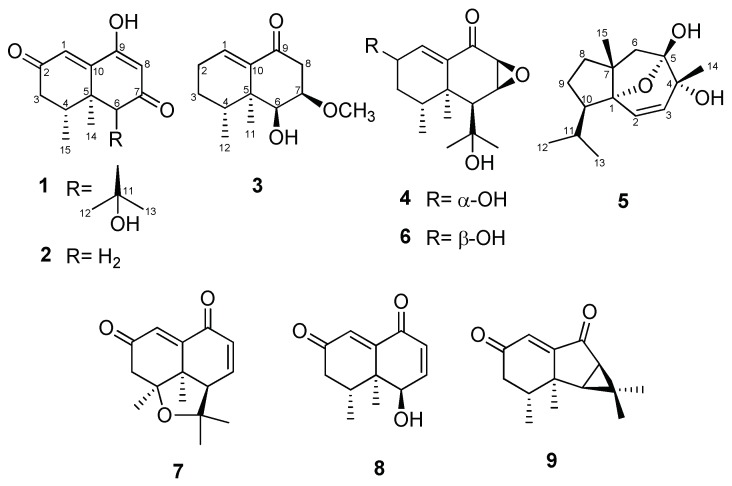
Structures of compounds **1**–**9**.

The relative configuration of compound **1** was determined by the analysis of ^1^H-NMR *J-*values and NOESY data. H-4 was assigned an axial (or pseudoaxial) orientation based on a large *trans*-diaxial coupling (*J* = 14.2 Hz) detected for it with H_ax_-3. With this orientation, NOESY correlations of H-6 with H_3_-14, H-6 with H_3_-15, and H_3_-14 with H_3_-15 indicated that these protons are on the same face of the naphthalenone ring. This was also supported by the NOESY correlation of H-4 with H_3_-13, which suggested that these protons are on the opposite face of the ring. Therefore, the structure of **1** was determined to be that shown in Figure 1, and it was named kanshone L.

Kanshone M (**2**) was isolated as a yellowish oil. Analysis of NMR (Table 1) and HRESIMS (C_12_H_14_O_3_) data revealed that the structure of **2** is identical to that of **1** except for the absence in **2** of the isopropyl alcohol group at the C-6 position in **1**. The planar structure of compound **2** was established through the detailed analysis of 2D NMR data.

As was done for compound **1**, the relative configuration of compound **2** was determined by analysis of ^1^H-NMR *J-*values and NOESY data. The NOESY correlations of H-6 with H_3_-14, H-6 with H_3_-15, and H_3_-14 with H_3_-15 suggested that the relative configurations of C-4, C-5, and C-6 are analogous to those in compound **1**. From the analysis of these data, the relative configuration of compound **2** was determined to be that shown in Figure 1, and it was named kanshone M.

7-Methoxydesoxonarchinol (**3**) was obtained as a colorless gum. Based on the analysis of ^1^H and ^13^C-NMR data (Table 2) along with HRESIMS data, its molecular formula was determined to be C_13_H_20_O_3_ (with four unsaturations). The ^1^H-NMR spectrum (Table 2) of this substance displayed characteristic signals of one olefinic proton (δ 6.75 (H-1)), a methoxy proton (δ 3.42 (H-7-OCH_3_)), two methine protons (δ 4.07 (H-6) and 3.76 (H-7)), and two methyl protons (δ 0.94 (H-12) and 0.84 (H-11)). The ^13^C-NMR and DEPT data indicated the presence of a carbonyl carbon (δ 199.6 (C-9)), an olefinic methine carbon (δ 138.2 (C-1)), a sp^2^ quaternary carbon (δ 140.7 (C-10)), a methoxy carbon (δ 56.4 (7-OCH_3_)), a sp^3^ quaternary carbon (δ 40.7 (C-5)), three sp^3^ methine carbons (δ 75.6 (C-7), 70.0 (C-6), and 31.1 (C-4)), three methylene carbons (δ 40.5 (C-8), 25.8 (C-2), and 25.7 (C-3)), and two methyl carbons (δ 19.3 (C-11) and 15.2 (C-12)). These results suggested that 7-methoxydesoxonarchinol (**3**) is structurally similar to the nardosinone-type sesquiterpenoid of desoxonarchinol A found in our previous study [5]. However, in the ^1^H-NMR spectrum of 7-methoxydesoxonarchinol (**3**) the signals corresponding to the two olefinic protons (H-7 and H-8) observed in desoxonarchinol A were not observed. On the other hand, an oxygenated methine proton (H-7), a methoxy proton (7-OCH_3_), and a methylene proton (H-8) were observed in the ^1^H-NMR spectrum. The positions of the new methylene group and methoxy group were confirmed by analysis of COSY and HMBC data. The COSY correlations of H-7 with H-6 and H_2_-8, along with the HMBCs of H-6 with C-7, C-8, and C-10, H_2_-8 with C-6, C-7, and C-9, and H-7 with 7-OCH_3_ confirmed that the structure of **3** was that shown in Figure 1. The relative configuration of 7-methoxydesoxonarchinol (**3**) was determined based on its NOESY spectrum. The NOESY correlations of its H_3_-11 with H-6, H-7, H-8α, and H_3_-12 indicated that these protons are all on the same α-face of this compound. Therefore, the structure of 7-methoxy-desoxo-narchinol (**3**) was determined to be that shown in Figure 1.

Kanshone N (**4**) was obtained as a colorless gum. Based on the analysis of ^1^H and ^13^C-NMR data (Table 2) along with HRESIMS data, its molecular formula was determined to be C_15_H_22_O_4_ (with five unsaturations). The ^1^H-NMR spectrum (Table 2) displayed signals of one olefinic proton (δ 6.76 (H-1)), three methine protons (δ 4.36 (H-2), 3.62 (C-7), and 3.38 (C-8)), and four methyl protons (δ 1.48 (H-12), 1.35 (H-13), 1.06 (H-14), and 0.98 (H-15)). The ^13^C-NMR and DEPT data and HSQCs indicated the presence of a carbonyl carbon (δ 193.8 (C-10)), an olefinic carbon (δ 140.5 (C-1)), a sp^2^ quaternary carbon (δ 140.7 (C-10)), two sp^3^ quaternary carbons (δ 75.3 (C-11) and 43.6 (C-5)), five sp^3^ methine carbons (δ 67.5 (C-2), 57.2 (C-8), 53.7 (C-7), 50.0 (C-6), and 31.6 (C-4)), a methylene carbon (δ 36.4 (C-3)), and four methyl carbons (δ 1.49 (C-12), 1.35 (C-13), 1.08 (C-14), and 0.99 (C-15)). Further analyses of 2D NMR data, including COSY and HMBC, suggested that kanshone N (**4**) has the same planar structure as kanshone D (**6**) (Figure 1) [19]. The relative configuration of **4** was determined based on NOESY and *J* values in the ^1^H-NMR data. In the ^1^H-NMR spectrum, the difference in *J* values between H-1 and H-2 was similar to that in the previously reported diastereomeric relationship between kanshone J and kanshone K, which have opposite configurations at the hydroxylated carbon C-2 [5]. In the case of kanshones J and K, the configurations of these compounds were determined through the comparison of *J* values and NOESY spectrums. The olefinic protons of kanshone J and kanshone D (**6**) were also previously determined to have high *J* values at δ 7.49 (H-1, dd, *J* = 5.3, 1.3 Hz) and δ 6.84 (H-1, d, *J* = 5.7 Hz), while kanshone K and kanshone N (**4**) were determined to have low *J* values at δ 7.50 (H-1, br dd, *J* = 1.8 Hz) and δ 6.77 (H-1, br t, *J* = 1.7 Hz). Additionally, the *J* values of the oxygenated methine protons of kanshone J and kanshone D (**6**) were assigned at δ 4.58 (H-2, br dd, *J* = 5.3, 4.3 Hz) and δ 4.25 (H-2, m), while those of kanshone K and kanshone N (**4**) were observed to be higher, at δ 4.74 (H-2, ddd, *J* = 10.3, 6.2, 1.8 Hz) and δ 4.36 (H-2, ddd, *J* = 10.5, 5.6, 1.7 Hz). H-2 was further assigned an axial (or pseudoaxial) orientation due to the detection of a large *trans*-diaxial coupling (*J* = 10.5 Hz) with H_ax_-3. The NOESY correlations of H-6 with H_3_-14 and of H-8 with H_3_-14 indicated that these protons are on the same face of the molecule. On the other hand, the NOESY correlation of H-2 with H_3_-13 suggested that these protons were oriented towards the same face of the structure. Therefore, we suggest that the methine proton (H-2) of kanshone N is on the β face of the structure, as shown in Figure 1.

Nardosdaucanol (**5**) was obtained as a yellow gum. Based on the analysis of ^1^H and ^13^C-NMR data (Table 3) along with HRESIMS data, its molecular formula was determined to be C_15_H_24_O_3_ (with four unsaturations). The ^1^H-NMR data for compound **5** (DMSO-*d_6_*) suggested the presence of two olefinic protons (δ 5.90 (H-2) and 6.03 (H-8)) and four methyl protons (δ 1.49 (H-14), 0.90 (H-12), 0.85 (H-15), and 0.847 (H-13)). The ^13^C-NMR and DEPT data also indicated the presence of two olefinic carbons (δ 131.84 (C-3) and 131.77 (C-2)), four sp^3^ quaternary carbons (δ 106.1 (C-5), 92.6 (C-1), 69.9 (C-4), and 55.4 (C-7)), two sp^3^ methine carbons (δ 52.9 (C-11) and 28.9 (C-12)), three methylene carbons (δ 45.7 (C-6), 39.2 (C-8), and 30.6 (C-9)), and four methyl carbons (δ 23.3 (C-12), 21.8 (C-15), 21.5 (C-14), and 20.5 (C-13)). This information accounts for one degree of unsaturation, and thus suggests that compound **5** must be a tricyclic sesquiterpenoid with two hydroxy groups. One-bond carbon-proton correlations were determined through HMQC analysis. In turn, COSY correlations from H-8 to H_3_-13 indicated the presence of a spin system corresponding to C8-C9-C10-C11-C12/C13. Furthermore, COSY correlations from H-2 to H-3 with the *J*-value (*J* = 9.7 Hz) indicative of mutually coupled olefinic protons suggested the presence of a *cis* olefin unit. The nature of the connections among the remaining units were established through the analysis of HMBC data. The HMBCs of H-9 with C-7 and of H_3_-15 with C-7 and C-8 indicated that C-7 was connected with C-8 and C-15. In addition, HMBCs of H_3_-15 with C-1, H-8 with C-1, H-9 with C-1, and H-10 with C-1 indicated that C-1 was connected to C-7 and C-10, establishing the cyclopentane moiety of compound **5**. The HMBCs of H-2 with C-1 and H-3 with C-1 established the connection of C1-C2/C3. Furthermore, HMBCs of H-2 with C-4 and of H-3 with C-4 and C-5 indicated the connection of C2-C3-C4-C5. Additionally, the methyl carbon, C-14, was determined to be connected to C-4 based on the HMBCs of H_3_-14 with C-3, C-4, and C-5. The HMBCs of H-6 with C-4, C-5, C-7, C-8, and C-15 indicated the connection of C4-C5-C6-C7, establishing the cycloheptene moiety of compound **5**. Two hydroxyl groups (4-OH and 5-OH) were located at C-4 and C-5 based on the consideration of chemical shifts and HMBCs of 4-OH with C-3, C-4, C-5, and C-14, and of 5-OH with C-4, C-5, and C-6. Finally, consideration of the molecular formula of the compound indicated that the compound must possess an ether group in a daucane structure. It was thus determined that the ether bridge likely connects positions 1 and 5 based on consideration of chemical shifts [23] and the fact that these two positions were the only remaining sites to be connected in the molecule. Therefore, the planar structure of compound **5** was identified as a new daucane-type sesquiterpenoid, as shown in Figure 1.

The relative configuration of compound **5** was determined by the analysis of NOESY spectrum data. NOESY correlations of H_3_-14 with H-6β and H_3_-15, H_3_-15 with H-6β, H-8β, and H_3_-14, and H-9β with H-11 and H_3_-13 indicated that these protons are on the same face of the azulene ring. In addition, the NOESY correlation of H-2 with H_3_-15 suggested that these protons are on the same face of the central ring, and thus the overall relative configuration of **5** was suggested to be that shown in Figure 1.

In addition, four known metabolites were isolated in this study. Based on the comparisons of their MS and NMR data with those previously reported in the literature, they were identified as kanshone D (**6**) [24], nardosinanone G (**7**) [25], narchinol A (**8**) [26], and nardoaristolone B (**9**) [27], respectively. Narchinol A (**8**) was previously reported to have protective effects against cardiomyocyte injury induced by hydrogen peroxide in neonatal rats [25]. However, the biological effects of the other compounds isolated have not previously been reported.

### 2.2. Effects of Compounds ***1**–**9*** on Nitrite and PGE_2_ Production in LPS-Stimulated BV2 Microglial Cells

Neurodegeneration, a progressive inflammatory disease, is closely associated with the overexpression of inflammatory mediators and cytokines, such as iNOS, COX-2 proteins, NO, PGE_2_, IL-1β, IL-12, IL-10, and TNF-α [28,29,30]. In this study, we first assessed the cytotoxic effects of the nine isolated compounds in LPS-induced BV2 microglial cells using MTT assays, and found that the viability of BV2 microglial cells was not significantly affected by compounds **1**–**9** in doses of 10.0 and 80.0 μM (data not shown). Upon stimulation with LPS, the production of NO and PGE_2_ in the cells increased; however, pretreatment of BV2 microglial cells with compounds **3**, **4**, and **8** reduced the production of NO and PGE_2_ (Table 4).

### 2.3. Effects of Compounds ***3***, ***4***, *and* ***8*** on the Expression of mRNA of the Pro-inflammatory Cytokines IL-10, IL-12, IL-1β, and TNF-α in LPS-Stimulated BV2 Microglial Cells

**Figure 2:**
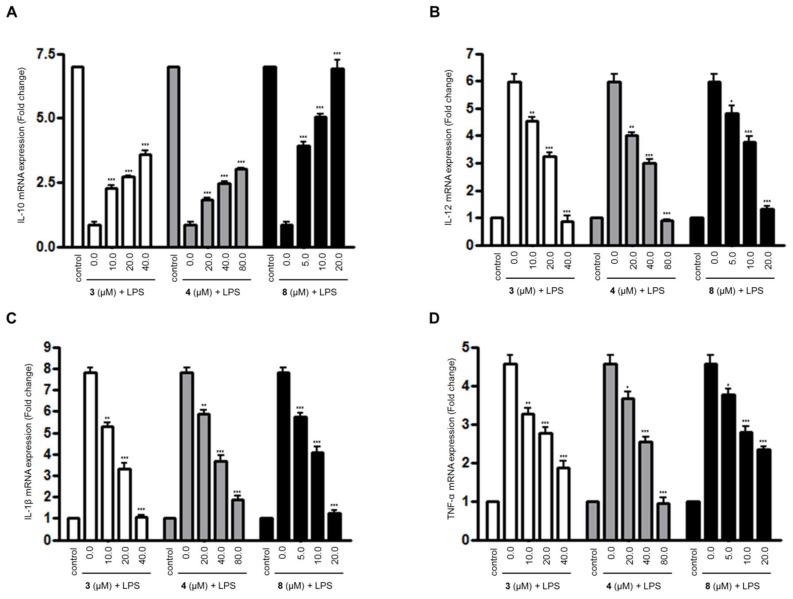
The effects of compounds **3**, **4**, and **8** on the expression of (**A**) interleukin (IL)-10, (**B**) IL-12, (**C**) IL-1β, and (**D**) tumor necrosis factor (TNF)-α mRNA in lipopolysaccharide (LPS)-stimulated BV2 microglial cells. (A–D) The cells were pretreated for 3 h with the indicated concentrations of compounds **3**, **4**, and **8,** and then stimulated for 6 h with LPS (1.0 μg/mL). The data are presented as the means ± SD of three experiments. The significance of the comparison against the LPS-treated group is indicated as follows: ^*^ *p* < 0.05; ^**^ *p* < 0.01; ^***^ *p* < 0.001. “+” is treated.

For compounds **3**, **4**, and **8**, which were the compounds that inhibited the overproduction of NO and PGE_2_ in LPS-treated BV2 microglial cell, we further evaluated their effects on the expression of inflammatory cytokines’ mRNA in LPS-treated BV2 microglial cells using quantitative real-time reverse transcriptase polymerase chain reaction (PCR) (Figure 2). As shown in Figure 2, compounds **3**, **4**, and **8** inhibited the expression of the mRNA of pro-inflammatory cytokines, such as IL-12, IL-1β, and TNF-α in a dose-dependent manner. In addition, compounds **3**, **4**, and **8** were shown to increase the expression of anti-inflammatory cytokine IL-10 mRNA in the cells treated with LPS.

### 2.4. Effects of Compounds ***3***, ***4,*** *and* ***8*** on iNOS and COX-2 Protein Expression in LPS-Stimulated BV2 Microglial Cells

**Figure 3:**
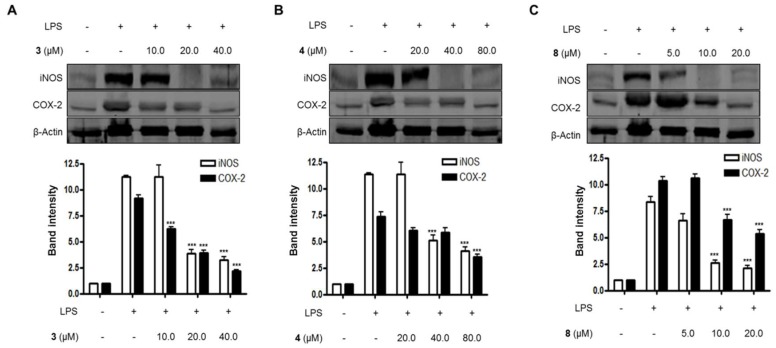
The effects of compounds (**A**) **3**, (**B**) **4**, and (**C**) **8** on the expression of iNOS and COX-2 proteins in lipopolysaccharide (LPS)-stimulated BV2 microglial cells. (A–C) The cells were pretreated for 3 h with the indicated concentrations of compounds **3**, **4**, and **8,** and then stimulated for 24 h with LPS (1.0 μg/mL). The data are presented as the means ± SD of three experiments. The band intensity was quantified by densitometry and normalized to the intensity of the β-actin band; lower panel, summarized bar graphs show band intensity presented as ratio of targeting protein over β-actin. The significance of the comparison against the LPS-treated group is indicated as follows: ^***^ *p* < 0.001. “+” is treated, while “-“ is not treated.

To investigate the effects of compounds **3**, **4**, and **8** on the expression of iNOS and COX-2 proteins in LPS-induced BV2 microglial cells, BV2 microglial cells were challenged with LPS (1 μg/mL) in the presence or absence of compounds **3**, **4**, and **8** at non-cytotoxic concentrations, and then the levels of iNOS and COX-2 protein expressed were measured. As shown in Figure 3, protein expression levels of iNOS and COX-2 in the BV2 cells were significantly up-regulated in response to LPS, while compounds **3**, **4**, and **8** suppressed iNOS and COX-2 protein expression in LPS-treated cells in a dose-dependent manner.

### 2.5. Effects of Compounds ***3***, ***4***, *and* ***8*** on IκB-α Levels and NF-κB Nuclear Translocation in LPS-Stimulated BV2 Microglial Cells

**Figure 4:**
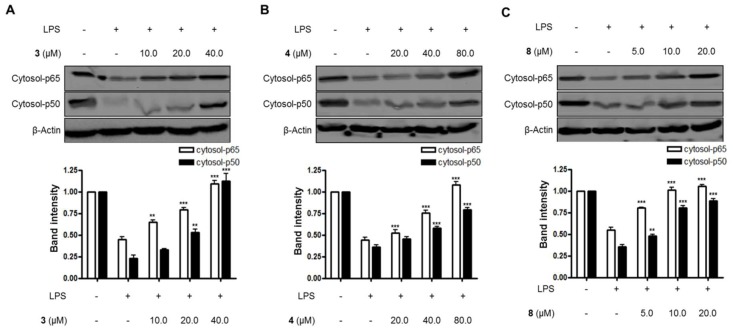
The effects of compounds (**A**) **3**, (**B**) **4**, and (**C**) **8** on LPS-induced NF-κB (p65/p50) expression in the cytosol of BV2 microglial cells. (A–C) The cells were pretreated for 3 h with the indicated concentrations of compounds **3**, **4**, and **8,** and then stimulated for 1 h with LPS (1.0 μg/mL). The data are presented as the means ± SD of three experiments. The band intensity was quantified by densitometry and normalized to the intensity of the β-actin band; lower panel, summarized bar graphs show band intensity presented as ratio of targeting protein over β-actin. The significance of the comparison against the LPS-treated group is indicated as follows: ^**^ *p* < 0.01; ^***^ *p* < 0.001. “+” is treated, while “-“ is not treated.

**Figure 5:**
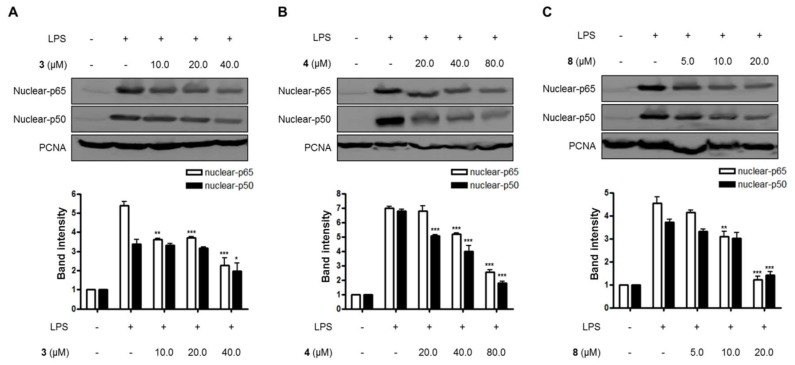
The effects of compounds (**A**) **3**, (**B**) **4**, and (**C**) **8** on LPS-induced NF-κB (p65/p50) expression in nuclear in BV2 microglial cells. (A–C) The cells were pretreated for 3 h with the indicated concentrations of compounds **3**, **4**, and **8,** and then stimulated for 1 h with LPS (1.0 μg/mL). The data are presented as the means ± SD of three experiments. The band intensity was quantified by densitometry and normalized to the intensity of the proliferating cell nuclear antigen (PCNA) band; lower panel, summarized bar graphs show band intensity presented as ratio of targeting protein over PCNA. The significance of the comparison against the LPS-treated group is indicated as follows: ^*^ *p* < 0.05; ^**^ *p* < 0.01; ^***^ *p* < 0.001. “+” is treated, while “-“ is not treated.

Lastly, the effects of compounds **3**, **4**, and **8** on the NF-κB pathway and the translocation of NF-κB in LPS-induced BV2 microglial cells were evaluated in this study. As shown in Figure 4 and Figure 5, compounds **3**, **4**, and **8** blocked the translocation of NF-κB dimers (p65/p50) into the nuclei of LPS-induced BV2 microglial cells.

**Figure 6:**
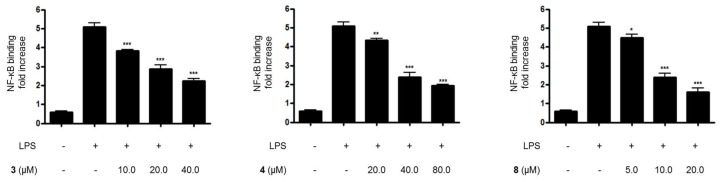
The effects of compounds (**A**) **3**, (**B**) **4**, and (**C**) **8** on LPS-induced NF-κB binding in BV2 microglial cells. (A–C) The cells were pretreated for 3 h with the indicated concentrations of compounds **3**, **4**, and **8,** and then stimulated for 1 h with LPS (1.0 μg/mL). The data are presented as the means ± SD of three experiments. The significance of the comparison against the LPS-treated group is indicated as follows: ^*^ *p* < 0.05; ^**^ *p* < 0.01; ^***^ *p* < 0.001. “+” is treated, while “-“ is not treated.

**Figure 7:**
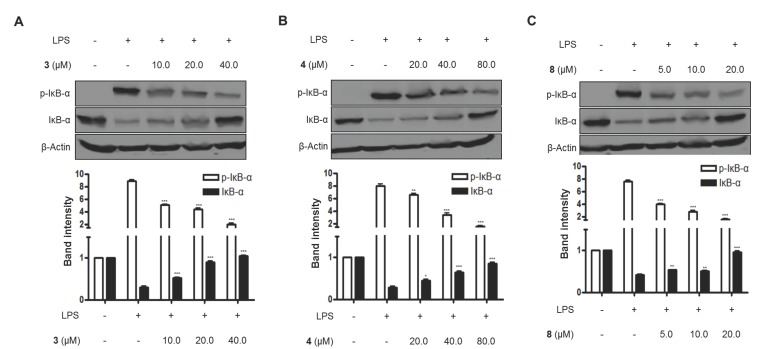
The effects of compounds (**A**) **3**, (**B**) **4**, and (**C**) **8** on LPS-induced IκB-α phosphorylation and degradation in BV2 microglial cells. (A–C) The cells were pretreated for 3 h with the indicated concentrations of compounds **3**, **4**, and **8,** and then stimulated for 1 h with LPS (1.0 μg/mL). The data are presented as the means ± SD of three experiments. The band intensity was quantified by densitometry and normalized to the intensity of the β-actin band; lower panel, summarized bar graphs show band intensity presented as ratio of targeting protein over β-actin. The significance of the comparison against the LPS-treated group is indicated as follows: ^*^ *p* < 0.05; ^**^ *p* < 0.01; ^***^ *p* < 0.001. “+” is treated, while “-“ is not treated.

Furthermore, when BV2 cells were treated with LPS alone, the DNA binding activity of NF-κB was remarkably increased. However, pretreatment with compounds **3**, **4**, and **8** at 3 h before the 1-h induction with LPS decreased NF-κB binding activity in a dose-dependent manner (Figure 6). Moreover, compounds **3**, **4**, and **8** were evaluated for their inhibitory effects on IκB-α phosphorylation and degradation. IκB-α was degraded after the exposure of BV2 microglial cells to LPS for 1 h. However, pretreatment with compounds **3**, **4**, and **8** significantly inhibited the phosphorylation of p-IκB-α in LPS-stimulated BV2 microglial cells (Figure 7). IκB-α in microglial cells was shown to be phosphorylated and degraded following LPS treatment (1.0 μg/mL). However, pretreatment with compounds **3**, **4**, and **8** for 3 h at concentrations ranging from 5.0 to 80.0 μM significantly inhibited the LPS-induced phosphorylation and degradation of IκB-α.

## 3. Experimental Section

### 3.1. General Information

Optical rotations were recorded using a Jasco P-1020 polarimeter (Jasco, Easton, MD, USA). 1D and 2D NMR spectra were recorded in chloroform-*d*, pyridine-*d*_5_, DMSO-*d*_6_, and MeOH-*d*_4_ using a JNM ECP-400 spectrometer (400 MHz for ^1^H and 100 MHz for ^13^C (JEOL, Tokyo, Japan). Additionally, ^1^H, ^13^C, COSY, HMQC, HMBC, and NOESY data were obtained using standard JEOL pulse sequences. Electrospray ionisation mass spectrometry (ESIMS) data were obtained at Korea University (Seoul, Korea) using a quadrupole time-of-flight mass spectrometer (Q-TOF) micro liquid chromatography–mass spectrometry (LC-MS/MS) instrument (Waters, Milford, MA, USA). Solvents used for extractions and flash column chromatography were of reagent grade and used without further purification. Solvents used for HPLC were of analytical grade. Flash column chromatography was carried out using octadecyl-functionalized C18 silica gel (YMC, Kyoto, Japan) and silica gel (Merck, Kenilworth, NJ, USA). HPLC separations were performed on a prep-C18 column (21.2 × 150 mm; 5 μm particle size) with a flow rate of 5 mL/min, and a semiprep-C18 column (10 × 250 mm; 5 μm particle size) with a flow rate of 3 mL/min. Dulbecco’s modified Eagle’s medium (DMEM), fetal bovine serum (FBS), and other tissue culture reagents were purchased from Gibco BRL Co. (Grand Island, NY, USA). Lipopolysaccharides from *Escherichia coli* 055:B5 were purchased from Sigma-Aldrich (St. Louis, MO, USA). All other chemicals were obtained from Sigma Chemical Co. (St. Louis, MO, USA). Primary antibodies, including mouse/goat/rabbit anti-COX-2 (sc-1745), anti-iNOS (sc-650), anti-β-actin (sc-47778), anti-IкB-α (sc-371), anti-phospho-IкB-α (sc-8404), anti-p50 (sc-7178), anti-p65 (sc-8008), and anti-proliferating cell nuclear antigen (PCNA) (sc-7907), and secondary antibodies were purchased from Santa Cruz Biotechnology (Dallas, TX, USA) [31].

### 3.2. Plant Material, Extraction, and Isolation

*Nardostachys jatamansi* was purchased from a standard commercial source (KwangMyungDang, Ulsan, Korea), and its identity was confirmed at the Korean Drug Test Laboratory (Seoul, Korea). Voucher specimens (WK-2016-03) were deposited at the College of Pharmacy Herbarium, Wonkwang University (Iksan, Korea). Extractions were performed on 5-kg samples of *Nardostachys jatamansi* (NJ) in methanol (24 L) with sonication for 2 h to obtain methanol extracts (663 g). The methanol extracts were dissolved in a mixture of H_2_O (8 L) and methanol (1 L), and sequentially partitioned with 18 L each of *n*-hexane, chloroform, ethyl acetate (EtOAc), and *n*-butanol. The 21.8 g EtOAc fraction (NJ(E)) obtained was subjected to silica gel column chromatography (CC) and eluted with hexane:EtOAc (3.5:1-1:0) and EtOAc:MeOH (99:1–20:80) to give 13 subfractions, NJ(E)-S1-S13. The subfraction NJ(E)-S(45)-7 was subjected to silica gel CC and eluted with hexane:EtOAc (3:1–0:1) to give 15 subfractions, NJ(E)-S(45)7-1-15. The subfraction NJ(E)-S(45)-7-11 was subjected to prep-HPLC and eluted with MeOH in H_2_O (0.1% formic acid) in gradients of 35–60% (0–30 min) to give **1** (10.3 mg, t_R_ = 17.5 min) and **2** (3.3 mg, t_R_ = 20.6 min). The hexane fraction (GSH-H, 307.6 g) was subjected to silica gel CC and eluted with a gradient of CH_3_Cl/MeOH (50:1–1:1) to give seven fractions, GSH-H1–GSH-H7. The subfraction GSH-H3 (72.7 g) was subjected to silica gel column chromatography and eluted with a gradient of hexane/EtOAc (20:1–1:1) to give six fractions, GSH-H3-1–GSH-H3-6, and then GSH-H3-5 (17.6 g) was further subjected to silica gel CC and eluted with a hexane/EtOAc gradient (10:1–1:1) to give thirteen subfractions, GSH-H3-5-1–10. The subfraction GSH-H3-5-7 (1.7 g) was eluted with a stepwise gradient of 50, 50, 60, 70, 80, 90, 100, and 100% (*v/v*) MeOH in H_2_O (500 mL each) to give eight subfractions, GSH-H3-5-7-1–8. The subfraction GSH-H3-5-7-3 was subjected to prep-HPLC and eluted with a gradient of 30–70% MeOH in H_2_O (0.1% formic acid) over 50 min to give **7** (1.6 mg, t_R_ = 27.4 min). The subfraction GSH-H3-5-7-5 (339.9 mg) was subjected to prep-HPLC and eluted with a gradient of 35–100% ACN in H_2_O (0.1% formic acid) over 50 min to give **9** (2.0 mg, t_R_ = 22.0 min). The subfraction GSH-H3-5-7-6 (568.3 mg) was subjected to prep-HPLC and eluted with a gradient of 40–100% MeOH in H_2_O (0.1% formic acid) over 50 min to give **5** (12.0 mg, t_R_ = 16.8 min). The subfraction GSH-H3-5-10 (741.5 mg) was subjected to silica gel CC and eluted with a gradient of CH_3_Cl/MeOH (20:1–1:1) to seven subfractions, GSH-H3-5-10-1–10. The subfraction GSH-H3-5-10-4 (212.6 mg) was subjected to prep-HPLC and eluted with a gradient of 45–100% MeOH in H_2_O (0.1% formic acid) over 25 min to give **8** (5.1 mg, t_R_ = 10.8 min). The subfraction GSH-H3-5-11b was subjected to prep-HPLC and eluted with a gradient of 40–100% MeOH in H_2_O (0.1% formic acid) over 50 min to give **3** (2.1 mg, t_R_ = 23.4 min). The subfraction GSH-H3-5-12 (393.2 mg) was eluted with a stepwise gradient of 60, 60, 60, 60, 80, and 100% (*v/v*) MeOH in H_2_O (500 mL each) to give seven subfractions, GSH-H3-5-12-1–7. The subfraction GSH-H3-5-12-1 (39.4 mg) was subjected to prep-HPLC and eluted with a gradient of 30–70% ACN in H_2_O (0.1% formic acid) over 50 min to give **4** (2.7 mg, t_R_ = 10.0 min) and **6** (4.4 mg, t_R_ = 12.6 min).

*Kanshone L* (**1**): yellowish oil; ${\left[\alpha \right]}_{D}^{25}$ + 29.4 (c 0.45, acetone); UV (MeOH) λ_max_ (log ε) 230 nm (4.0); ^1^H and ^13^C-NMR (400 and 100 MHz, acetone*-d_6_*, pyridine-*d_5_*) data, see Table 1; HRESIMS *m/z* 287.1241 [M + Na]^+^, calculated for C_15_H_20_O_4_Na, 287.1259. (Figures S1–S5).

*Kanshone M* (**2**): yellowish oil;$\;{\left[\alpha \right]}_{D}^{25}$ + 82.2 (c 0.30, acetone); UV (MeOH) λ _max_ (log ε) 270 nm (3.3); ^1^H and ^13^C-NMR (400 and 100 MHz, pyridine-*d_5_*) data, see Table 1; HRESIMS *m/z* 207.1003 [M + Na]^+^, calculated for C_12_H_15_O_3_, 207.1021. (Figures S6–S8).

*7-Methoxydesoxonarchinol* (**3**): colorless oil;$\;{\left[\alpha \right]}_{D}^{23}$ +76.7 (c 0.03, chloroform); UV (MeOH) λ _max_ (log ε) 242 nm (3.2); ^1^H and ^13^C-NMR (400 and 100 MHz, chloroform-*d*) data, see Table 2; HRESIMS *m/z* 247.1305 [M + Na]^+^, calculated for C_12_H_15_O_3_Na, 247.1310. (Figures S9–S11).

*Kanshone N* (**4**): colorless oil;$\;{\left[\alpha \right]}_{D}^{23}$ − 18.7 (c 0.10, chloroform); UV (MeOH) λ _max_ (log ε) 254 nm (3.3); ^1^H and ^13^C-NMR (400 and 100 MHz, chloroform-*d*) data, see Table 2; HRESIMS *m/z* 289.1418 [M + Na]^+^, calculated for C_15_H_22_O_3_Na, 289.1416. (Figures S12–S14).

*Nardosdaucanol* (**5**): yellowish oil;$\;{\left[\alpha \right]}_{D}^{23}$ − 63.9 (c 0.22, chloroform); UV (MeOH) λ _max_ (log ε) 210 nm (2.9); ^1^H and ^13^C-NMR (400 and 100 MHz, DMSO-*d_6_*, pyridine-*d_5_*) data, see Table 3; HRESIMS *m/z* 275.1618 [M + Na]^+^, calculated for C_15_H_24_O_3_Na, 275.1623. (Figures S15–S19).

### 3.3. Cell Culture and Viability Assay

BV2 microglial cells were obtained from Prof. Hyun Park at Wonkwang University (Iksan, Korea). BV2 microglial cells were maintained at 5 × 10^6^ cells/dish (5 × 10^5^ cells/mL) in 100-mm diameter dishes in DMEM supplemented with 10% (*v*/*v*) heat-inactivated FBS, penicillin G (100 units/mL), streptomycin (100 μg/mL), and L-glutamine (2 mM), and incubated at 37 °C in a humidified atmosphere containing 5% CO_2_. To determine cell viability, cells were plated in 96-well plates (2 × 10^4^ cells/well) and were incubated with 3-(4,5-dimethylthiazol-2-yl)-2,5-diphenyl-tetrazolium bromide (MTT, Sigma-Aldrich, Darmstadt, Germany) at a final concentration of 0.5 mg/mL for 4 h. The formazan salt formed was dissolved in acidified 2-propanol and the optical density of the solution was measured at 590 nm using a microplate reader (Bio-Rad, Hercules, CA, USA). The optical density of the formazan formed in control (untreated) cells was considered to represent 100% viability. The assay was independently repeated three times [31].

### 3.4. Determination of Nitrite (NO) Production

The nitrite concentration in the medium, which is an indicator of NO production, was measured using the Griess reaction. Three independent assays were performed. A 100-μL aliquot of the supernatant was mixed with an equal volume of Griess reagent (Solution A: 222488; Solution B: S438081; Sigma-Aldrich) and the absorbance of the mixture at 525 nm was determined using an enzyme-linked immunosorbent assay (ELISA) plate reader (model 680, Bio-Rad) [31].

### 3.5. PGE_2_ Assay

The level of PGE_2_ present in each sample was determined using a kit commercially available from R&D Systems (Minneapolis, MN, USA), following the manufacturer’s instructions. The assay was repeated independently three times. Briefly, BV2 microglial cells were cultured in 24-well plates, preincubated for 3 h in different concentrations of compounds, and then stimulated for 24 h with LPS. The cell culture supernatants were collected immediately after treatment and spun at 13,000× *g* for 2 min to remove particulate matter. The medium was then added to a 96-well plate precoated with affinity-purified polyclonal antibodies specific for PGE_2_. An enzyme-linked polyclonal antibody specific for PGE_2_ was added to the wells, left to react for 24 h, and then washed to remove any unbound antibody-enzyme reagent. After the addition of a substrate solution, the intensity of color produced at 450 nm, which was proportional to the amount of PGE_2_ present, was measured [31].

### 3.6. Quantitative Real-Time Reverse Transcriptase PCR (qRT-PCR)

Total RNA was isolated from the cells by using Trizol (Invitrogen, Carlsbad, CA, USA) according to the manufacturer’s recommendations, and then quantified spectrophotometrically at 260 nm. Total RNA (1 μg) was reverse transcribed using the High Capacity RNA-to-cDNA kit (Applied Biosystems, Carlsbad, CA, USA). The cDNA was then amplified with the SYBR Premix Ex Taq kit (TaKaRa Bio, Shiga, Japan) using a StepOnePlus Real-Time PCR system (Applied Biosystems). Briefly, each 20 μL reaction volume contained 10 μL of SYBR Green PCR Master Mix, 0.8 μM of each primer, and diethyl pyrocarbonate (DEPC)-treated water. The primer sequences were designed using PrimerQuest (Integrated DNA Technologies, Cambridge, MA, USA). The mouse primer sequences were 5′-CCA GAC CCT CAC ACT CAC AA-3′ (forward) and 5′-ACA AGG TAC AAC CCA TCG GC-3′ (reverse) for TNF-α; 5′-AAT TGG TCA TAG CCC GCA CT-3′ (forward) and 5′-AAG CAA TGT GCT GGT GCT TC-3′ (reverse) for IL-1β; 5′-GTA GAA GTG ATG CCC CAG GC-3′ (forward) and 5′-GAA ATC GAT GAC AGC GCC TC-3′ (reverse) for IL-10; and 5′-AGT GAC ATG TGG AAT GGC GT-3′ (forward) and 5′-CAG TTC AAT GGG CAG GGT CT-3′ (reverse) for IL-12. The optimum conditions for PCR amplification of the cDNA were established by following the manufacturer’s instructions, and the data were analyzed using Step One software (Step One Software 2.0, Applied Biosystems, Foster City, CA, USA). Values of the cycle number at the linear amplification threshold (Ct) for the endogenous control gene (GAPDH) and each target gene were recorded. The relative gene expression (target gene expression normalized to the expression of the endogenous control gene) was calculated using the comparative Ct method (2−ΔΔCt). The analysis was repeated independently three times [31].

### 3.7. Preparation of Cytosolic and Nuclear Fractions

BV2 and primary rat microglial cells were homogenized in M-PER™ Mammalian Protein Extraction Buffer (1:20, *w/v*) (Pierce Biotechnology, Rockford, IL, USA) containing freshly added protease inhibitor cocktail I (EMD Biosciences, San Diego, CA, USA) and 1 mM phenylmethyl-sulfonylfluoride (PMSF). The cytosolic fraction of the cells was prepared by centrifugation at 16,000× *g* for 5 min at 4 °C. The nuclear and cytoplasmic cell extracts were prepared with NE-PER^®^ nuclear and cytoplasmic extraction reagents (Pierce Biotechnology), respectively [32].

### 3.8. Western Blot Analysis

BV2 microglial cells were harvested and pelleted by centrifugation at 16,000 rpm for 15 min. The cells were then washed with phosphate-buffered saline and lysed in 20 mM Tris-HCl buffer (pH 7.4) containing a protease inhibitor mixture (0.1 mM phenylmethylsulfonylfluoride, 5 mg/mL aprotinin, 5 mg/mL pepstatin A, and 1 mg/mL chymostatin). The protein concentration was determined using a Lowry protein assay kit (P5626; Sigma-Aldrich). An equal amount of protein from each sample was resolved using 7.5% or 12% sodium dodecyl sulfate-polyacrylamide gel electrophoresis and then electrophoretically transferred onto a Hybond™ enhanced chemiluminescence nitrocellulose membrane (Bio-Rad). The membrane was blocked with 5% (*w*/*v*) skim milk before sequential incubation with the primary antibody (Santa Cruz Biotechnology) and the horseradish peroxidase-conjugated secondary antibody, followed by detection using enhanced chemiluminescence (Amersham Pharmacia Biotech, Piscataway, NJ, USA). The signal intensities were quantified using densitometric ImageJ software (National Institutes of Health, Bethesda, MD, USA). Molecular weight markers were used, as were the internal standards, β-actin and PCNA. Three independent membranes were analyzed to detect appropriate proteins. [32].

### 3.9. DNA Binding Activity of NF-κB

BV2 microglial cells were pre-treated for 3h with the indicated concentrations of cudratricusxanthone L prior to stimulating for 1 h with LPS (1 μg/mL). The DNA-binding activity of NF-κB in nuclear extracts was measured using the TransAM^®^ kit (Active Motif, Carlsbad, CA, USA) according to the manufacturer’s instructions. The assay was conducted three times independently [32].

### 3.10. Statistical Analysis

All data are expressed herein as the mean ± standard deviation (SD) values of at least three independent experiments. To compare three or more groups, one-way analyses of variance (ANOVAs) followed by Tukey’s multiple comparison tests were carried out. Data were analyzed using GraphPad Prism software, version 3.03 (GraphPad Software Inc., San Diego, CA, USA) [33].

## 4. Conclusions

In this study, five novel and four known compounds were isolated from *Nardostachys jatamansi*. Among the isolated compounds, two novel nardosinone-type (compounds **1** and **2**) and one novel daucane-type (compound **5**) sesquiterpenoids did not show anti-inflammatory effects. However, compounds **3**, **4,** and **8** displayed inhibitory effects on the overproduction of pro-inflammatory cytokines, such as IL-1β, IL-12, and TNF-α, as well as on pro-inflammatory mediators including NO and PGE_2_ in LPS-stimulated BV2 microglial cells. Moreover, we verified that these inhibitory effects were due to the ability of **3**, **4,** and **8** to inactivate the NF-κB pathway, blocking the translocation and binding of NF-κB and its dimers (p65/p50). It is also possible that the presence of an α,β-unsaturated carbonyl group in compound **8**, the most active compound found, contributed to its inhibitory effects, as was demonstrated by a number of previous studies related to Michael acceptor systems [34]. In addition, it was found that the modification of the nardosinone skeleton at the C-6 position may play an important role in the potency of anti-inflammatory effects, which was analogous with the results of previous studies [5,35]. This was observed through the replacement of the isopropyl alcohol group of compound **7** by the hydroxy group of compound **8**, which led to significantly increased activity.

## Figures and Tables

**Table 1:** NMR data for compounds **1** and **2**.

Position		1				2		
		*δ*_C_^a,c^	*δ*_H_^b,c^, mult (*J* in Hz)	*δ*_C_^a,d^	*δ*_H_^b,d^, mult (*J* in Hz)		*δ*_C_^a,d^	*δ*_H_^b,d^, mult (*J* in Hz)
1		124.7	6.53, s	125.5	7.22, s		125.6	7.08, s
2		198.0		198.9			198.7	
3	α	41.9	2.41, dd (17.2, 14.2)	42.5	2.50, m		42.5	2.40, br d (8.5)
	β		2.25, dd (17.2, 4.6)		2.50, m			2.40, br d (8.5)
4		33.5	3.29, ddq (14.2, 6.4, 4.6)	33.9	3.67, ddq (12.7, 6.4, 5.8)		39.8	2.11, ddd (8.5, 6.8, 2.0)
5		42.3		42.8			39.3	
6	α	65.8	2.57, s	66.8	3.07, s		48.7	2.73, d (15.6)
	β							2.42, d (15.6)
7		197.7		198.9			194.6 ^e^	
8		106.5	5.54, s	107.0	6.03, s		107.1	6.02, s
9		167.5		168.1			169.1 ^e^	
10		157.7		158.6			158.1 ^e^	
11		72.1		72.2			18.9	1.14, s
12		32.7	1.17, s	33.5	1.55, s		14.8	0.81, d (6.8)
13		26.8	1.23, s	28.0	1.57, s			
14		21.2	1.19, s	21.8	1.33, s			
15		16.1	1.08, d (6.4)	16.7	1.14, d (6.4)			

**^a^** Recorded at 100 MHz. ^b^ Recorded at 400 MHz. ^c^ Recorded in acetone-*d_6_*. ^d^ Recorded in pyridine-*d_5_*. ^e^ Determined based on HMBC data.

**Table 2:** NMR data for compounds **3** and **4**.

Position		3		4	
		*δ*_C_^a,c^	*δ*_H_^b,c^, mult (*J* in Hz)	*δ*_C_^a,c^	*δ*_H_^b,c^, mult (*J* in Hz)
1		138.2	6.75, dd, (4.9, 2.9)	140.4	6.76, br t (1.7)
2		25.8	2.22, m	67.4	4.36, ddd (10.5, 5.6, 1.7)
3	α	25.7	1.54, m	36.4	1.26, m
	β		1.54, m		1.83, dddd (12.4, 4.3, 2.9, 1.7)
4		31.1	2.28, ddq (13.0, 6.8, 3.7)	31.5	2.74, m
5		40.7		43.5	
6		70.0	4.07, d (2.4)	49.9	2.30, d (6.3)
7		75.6	3.76, ddd (10.5, 6.4, 2.4)	57.2	3.62, dd (6.3, 3.4)
8	α	40.5	2.81, dd (17.0, 6.4)	53.7	3.38, d (3.4)
	β		2.56, dd (17.0, 10.5)		
9		199.6		193.8	
10		140.7		140.7	
11		19.3	0.84, s	75.3	
12		15.2	0.95, d (6.8)	33.0^d^	1.48, s
13		56.4	3.42, s	28.1^d^	1.35, s
14				24.7	1.06, s
15				17.2	0.98, d (6.5)

^a^ Recorded at 100 MHz. ^b^ Recorded at 400 MHz. ^c^ Recorded in chloroform-*d*. ^d^ Determined based on HSQC data.

**Table 3:** NMR data for compound **5**.

Position		5			
		*δ*_C_^a,c^	*δ*_H_^b,c^, mult (*J* in Hz)	*δ*_C_^a,d^	*δ*_H_^b,d^, mult (*J* in Hz)
1		92.6		93.7	
2		131.77	5.90, d (9.7)	132.8	5.97, s ^*^
3		131.84	5.61, d (9.7)	132.9	5.97, s ^*^
4		69.9		71.2	
5		106.1		107.3	
6	α	45.7	1.79, d (13.3)	46.2	2.30, d (13.4)
	β		1.69, d (13.3)		1.97, d (13.4)
7		55.4		56.4	
8	α	39.2	1.81, m	40.0	2.14, m
	β		1.52, m ^*^		1.51, m
9	α	30.6	1.85, m	31.3	1.83, m
	β		1.52, m ^*^		1.52, m
10		52.9	1.76, m	53.7	1.97, m
11		28.9	1.63, m	29.7	1.44, m
12		23.3	0.90, d (6.4)	23.4	0.72, d (6.5)
13		20.5	0.84, d (6.4)	20.6	0.77, d (6.5)
14		21.5	1.08, s	21.9	1.48, s
15		21.8	0.85, s	22.0	0.92, s
4-OH			4.50, s		
5-OH			5.35, s		

**^a^** Measured at 100 MHz. ^b^ Measured at 400 MHz. ^c^ Measured in DMSO-*d*_6_. ^d^ Measured in pyridine-*d*_5_. ^*^ Overlapped.

**Table 4:** Inhibitory effects of compounds **1**–**9** on NO and PGE_2_ production.

Compound	NO Production Inhibitory Effects in BV2 Microglial Cells (IC_50_ = μM)	PGE_2_ Production Inhibitory Effects in BV2 Microglial Cells (IC_50_ = μM)
1	n.d.	n.d.
2	n.d.	n.d.
3	40.2 ± 2.0 ^*^	35.8 ± 1.8 ^*^
4	55.3 ± 2.8 ^*^	56.2 ± 2.8 ^*^
5	n.d.	n.d.
6	n.d.	n.d.
7	n.d.	n.d.
8	9.3 ± 0.5 ^*^	13.5 ± 0.7 ^*^
9	70.9 ± 3.5 ^*^	76.7 ± 3.8 ^*^

Each value represents the mean ± SD (*n = 3*). ^*^ *p* < 0.001, compared with the LPS group.

## Supplementary Materials

The following are available online, spectrum of compounds **1**–**5**.
