# Supplementary material for: Isolation of Novel Sesquiterpeniods and Anti-neuroinflammatory Metabolites from Nardostachys jatamansi
Source: Molecules. 2018 Sep 17;23(9):2367. doi: 10.3390/molecules23092367 (PMC6225174; doi:10.3390/molecules23092367)
Supplement: Supplementary file 1 [file molecules-23-02367-s001.pdf]

Supplementary Materials

Isolation of Novel Sesquiterpeniods and Anti-neuroinflammatory Metabolites from *Nardostachys jatamansi*

Chi-Su Yoon [1, 2, #], Dong-Cheol Kim [1, 2, #], Jin-Soo Park [1, #], Kwan-Woo Kim [1], Youn-Chul Kim [1], and Hyuncheol Oh[1, 2, *]

[1] College of Pharmacy, Wonkwang University, Iksan 54538, Republic of Korea; ycs1991@naver.com (C.-S.Y.); js9181515@naver.com (J.-S.P.); kimman07@hanmail.net (D.-C.K); swamp1@naver.com (K.-W.K); yckim@wku.ac.kr (Y.-C.K)

[2] Hanbang Cardio-Renal Syndrome Research Center, Wonkwang University, Iksan 54538, Republic of Korea

* Correspondence: hoh@wku.ac.kr (H.O.); Tel.: +82-63-850-6815 (H.O.); Fax: +82-63-852-8837 (H.O.)

[#] These authors contributed equally to this work.

List of Supporting Information

Figure S1. HRESIMS spectrum of compound **1**.

Figure S2. ^{1}H NMR (400 MHz, acetone-d_6) spectrum of compound **1**.

Figure S3. ^{13}C NMR (100 MHz, acetone-d_6) spectrum of compound **1**.

Figure S4. ^{1}H NMR (400 MHz, pyridine-d_5) spectrum of compound **1**.

Figure S5. ^{13}C NMR (100 MHz, pyridine-d_5) spectrum of compound **1**.

Figure S6. HRESIMS spectrum of compound **2**.

Figure S7. ^{1}H NMR (400 MHz, pyridine-d_5) spectrum of compound **2**.

Figure S8. ^{13}C NMR (100 MHz, pyridine-d_5) spectrum of compound **2**.

Figure S9. HRESIMS spectrum of compound **3**.

Figure S10. ^{1}H NMR (400 MHz, chloroform-d) spectrum of compound **3**.

Figure S11. ^{13}C NMR (100 MHz, chloroform-d) spectrum of compound **3**.

Figure S12. HRESIMS spectrum of compound **4**.

Figure S13. ^{1}H NMR (400 MHz, chloroform-d) spectrum of compound **4**.

Figure S14. ^{13}C NMR (100 MHz, chloroform-d) spectrum of compound **4**.

Figure S15. HRESIMS spectrum of compound **5**.

Figure S16. ^{1}H NMR (400 MHz, DMSO-d_6) spectrum of compound **5**.

Figure S17. ^{13}C NMR (100 MHz, DMSO-d_6) spectrum of compound **5**.

Figure S18. ^{1}H NMR (400 MHz, pyridine-d_5) spectrum of compound **5**.

Figure S19. ^{13}C NMR (100 MHz, pyridine-d_5) spectrum of compound **5**.

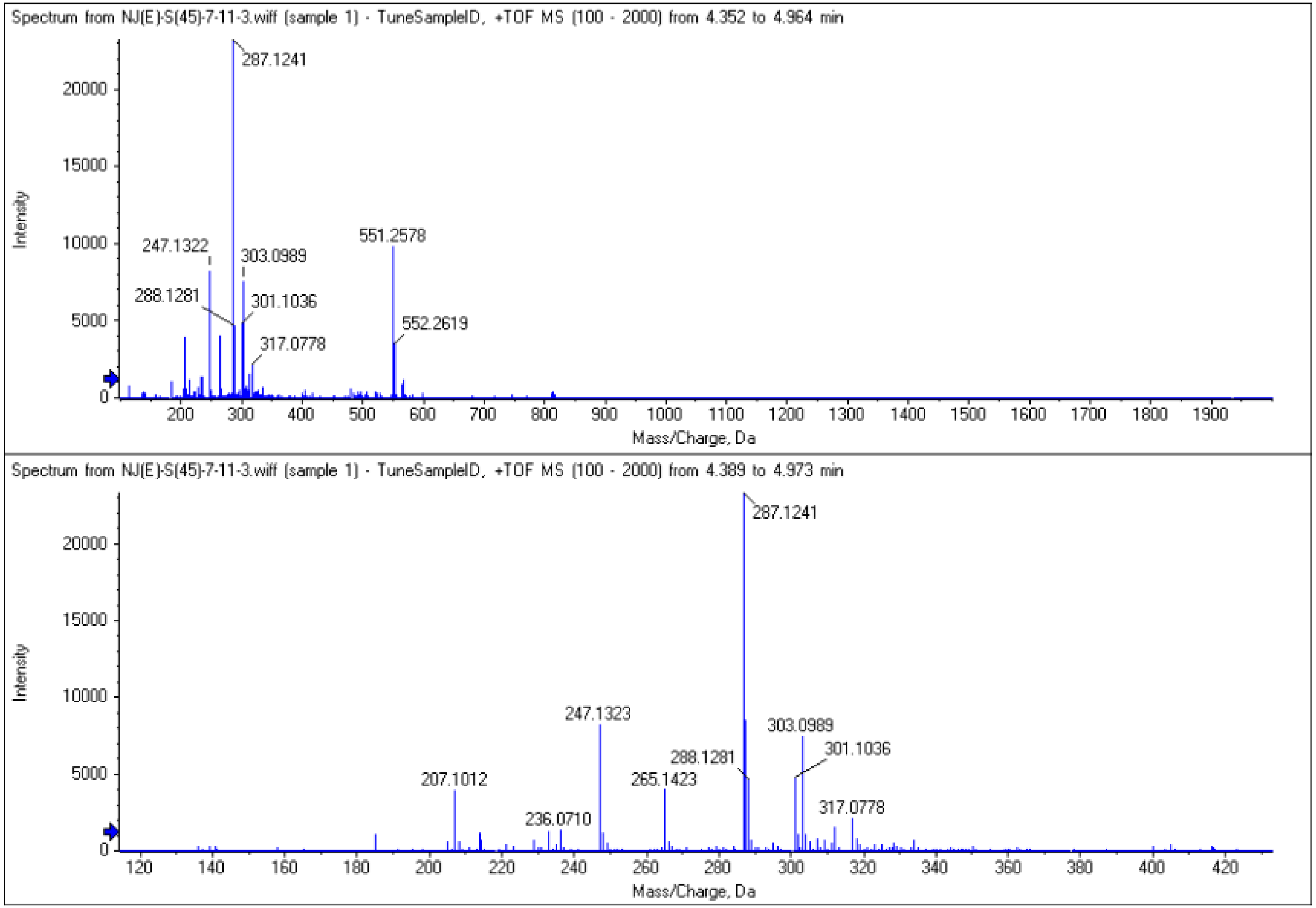

Figure S1. HRESIMS spectrum of compound **1**.

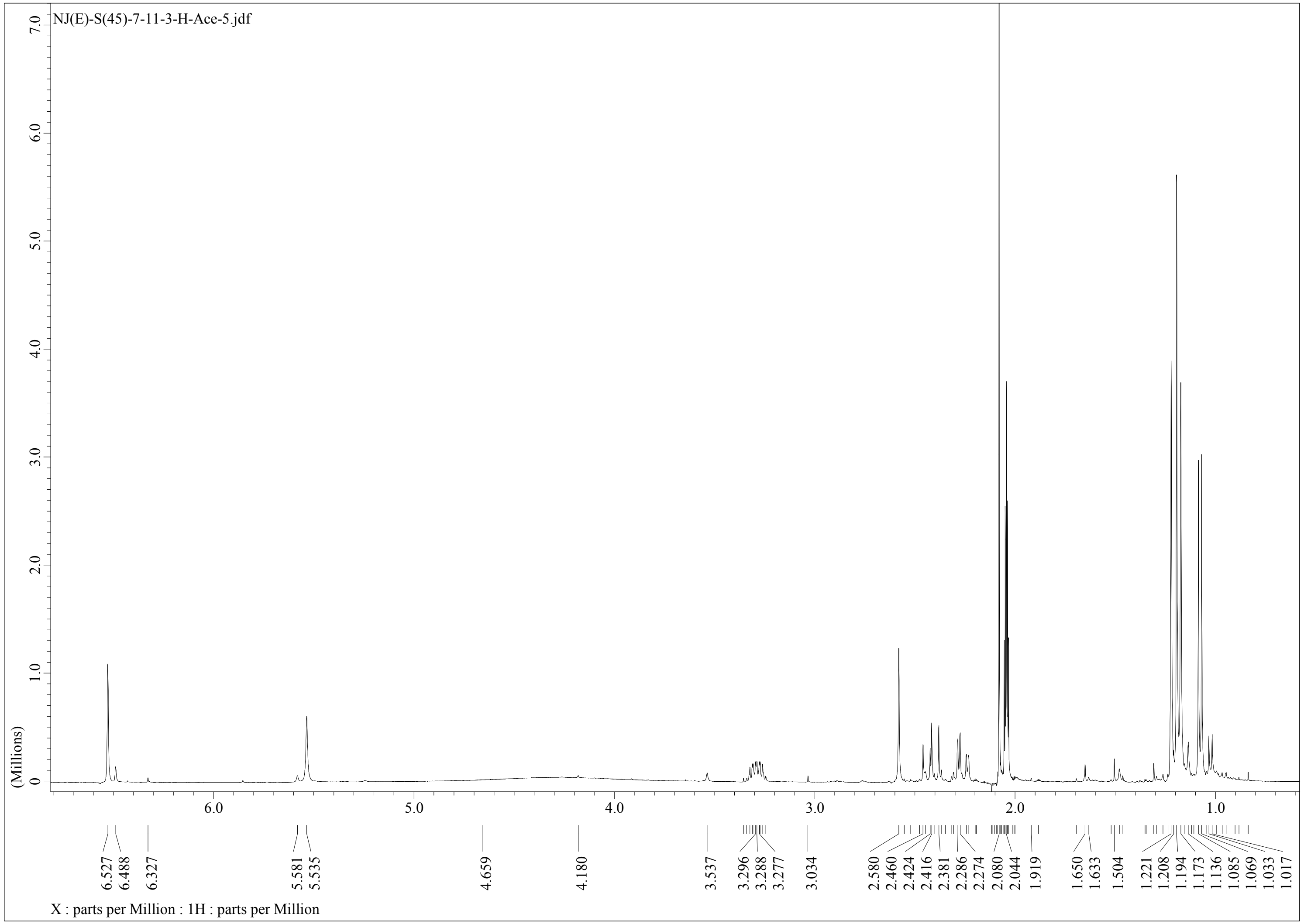

Figure S2. ^{1}H NMR (400 MHz, acetone-d_6) spectrum of compound **1**.

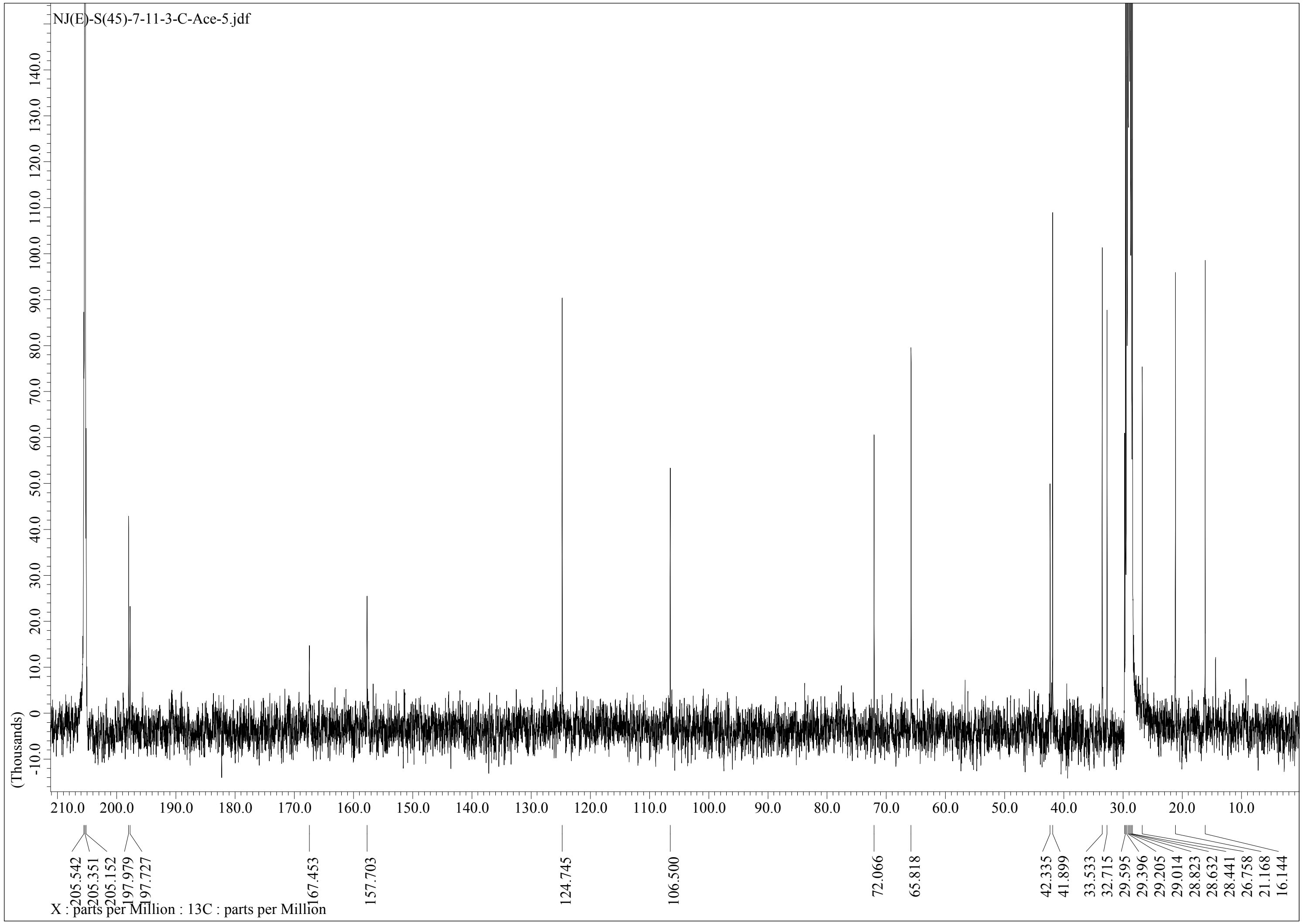

Figure S3. ^{13}C NMR (100 MHz, acetone-d_6) spectrum of compound **1**.

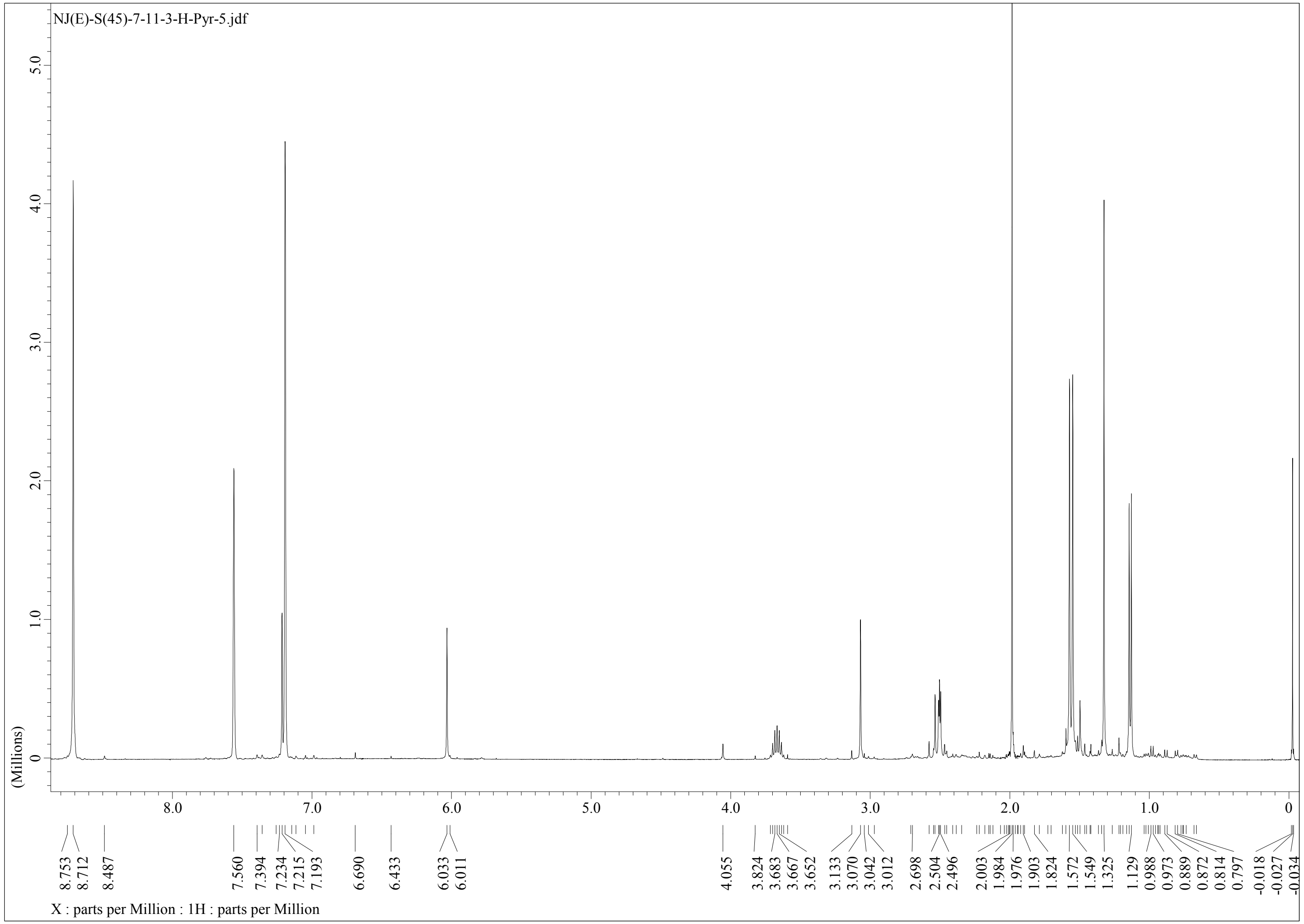

Figure S4. ^{1}H NMR (400 MHz, pyridine-d_5) spectrum of compound **1**.

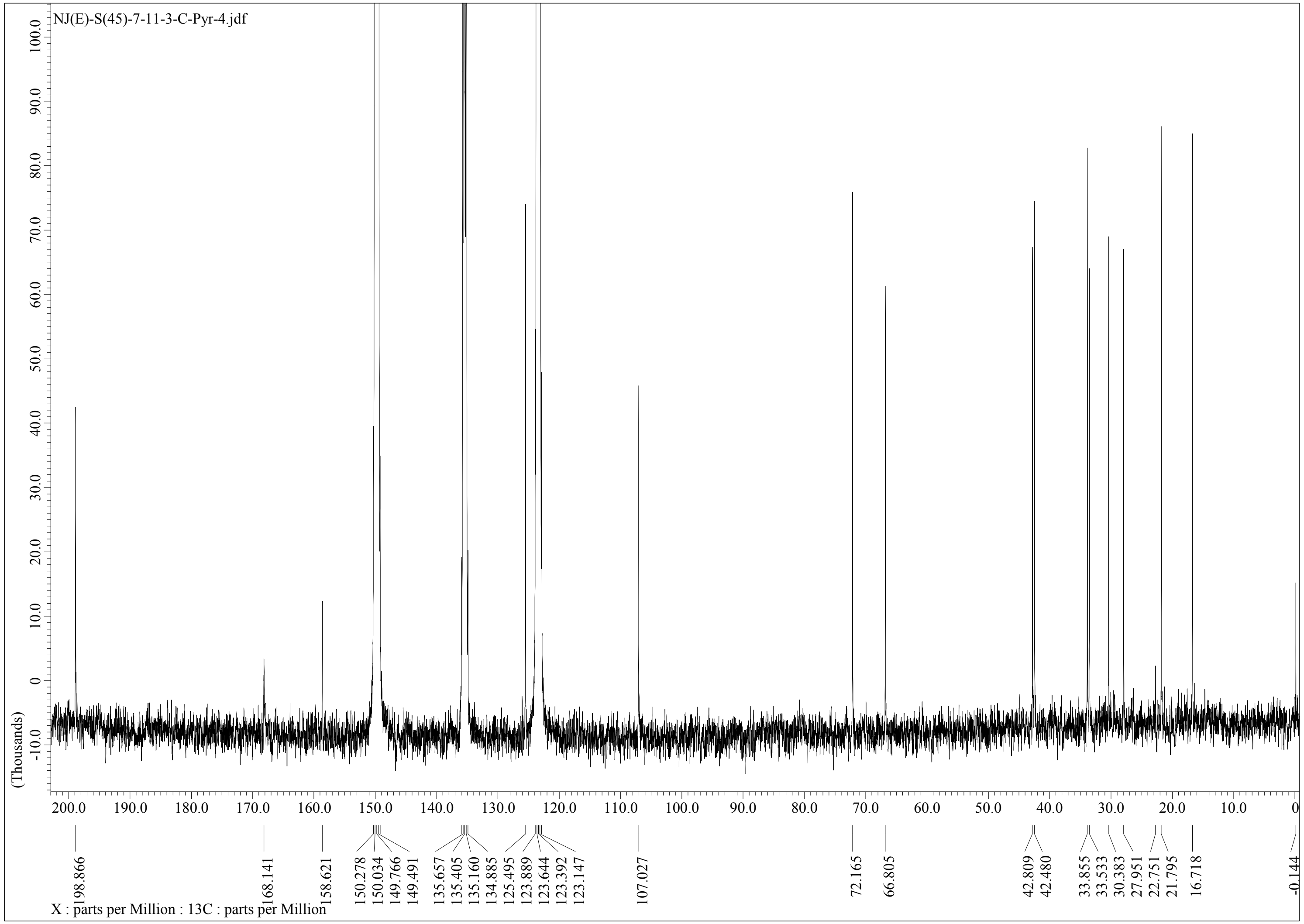

Figure S5. ^{13}C NMR (100 MHz, pyridine-*d*5) spectrum of compound **1**.

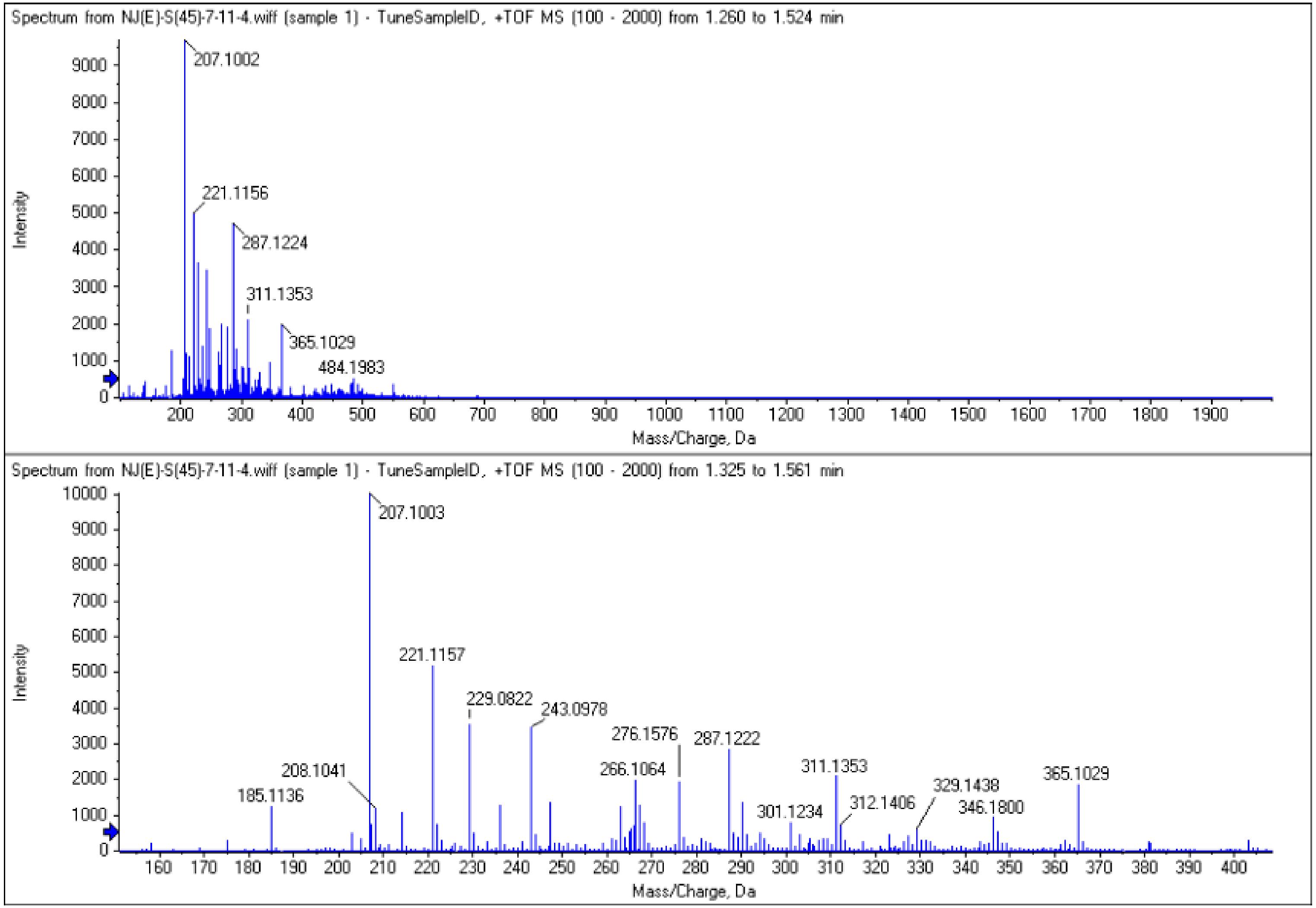

Figure S6. HRESIMS spectrum of compound **2**.

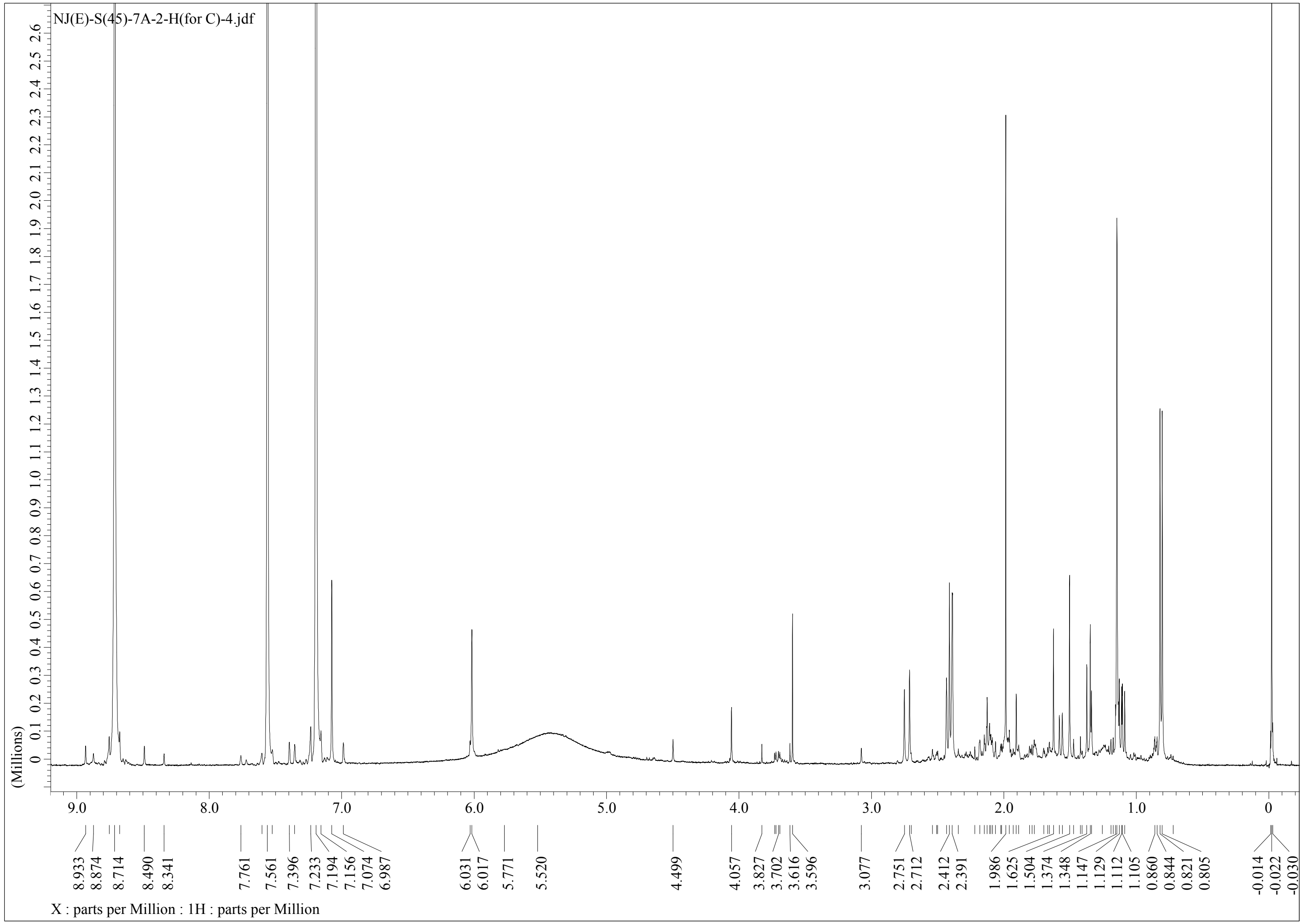

Figure S7. ^{1}H NMR (400 MHz, pyridine-d_5) spectrum of compound **2**.

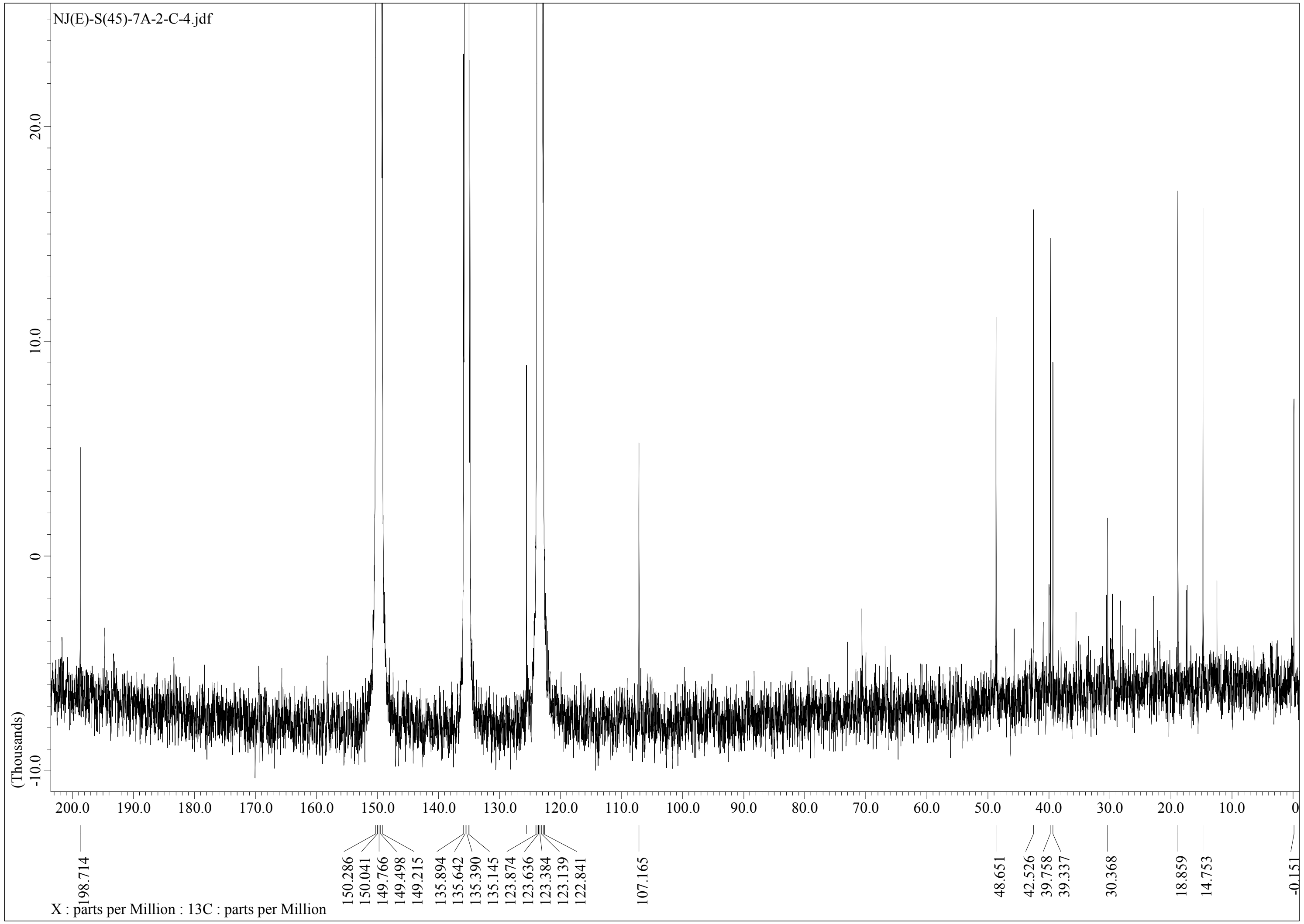

Figure S8. ^{13}C NMR (100 MHz, pyridine-d_5) spectrum of compound **2**.

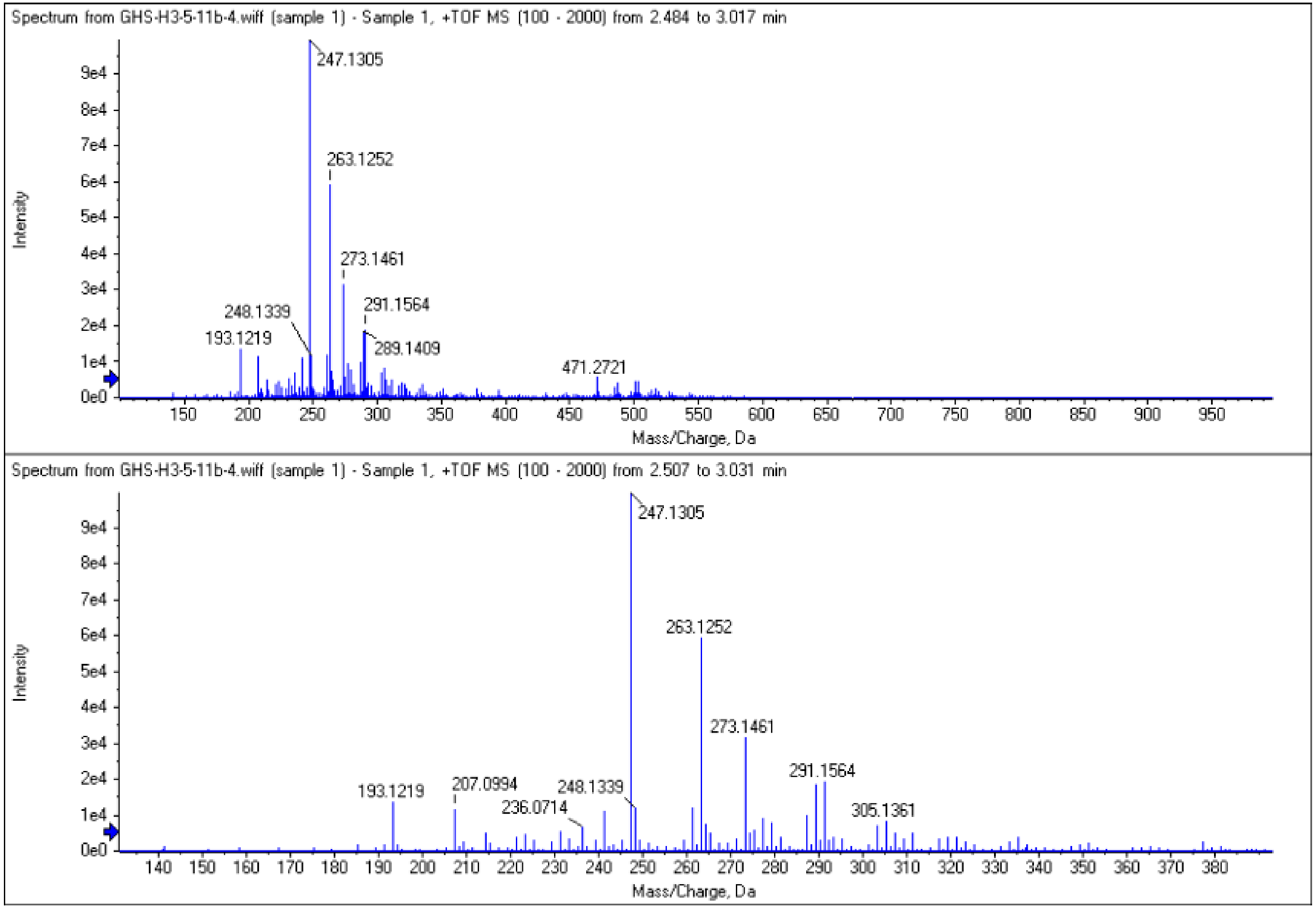

Figure S9. HRESIMS spectrum of compound **3**.

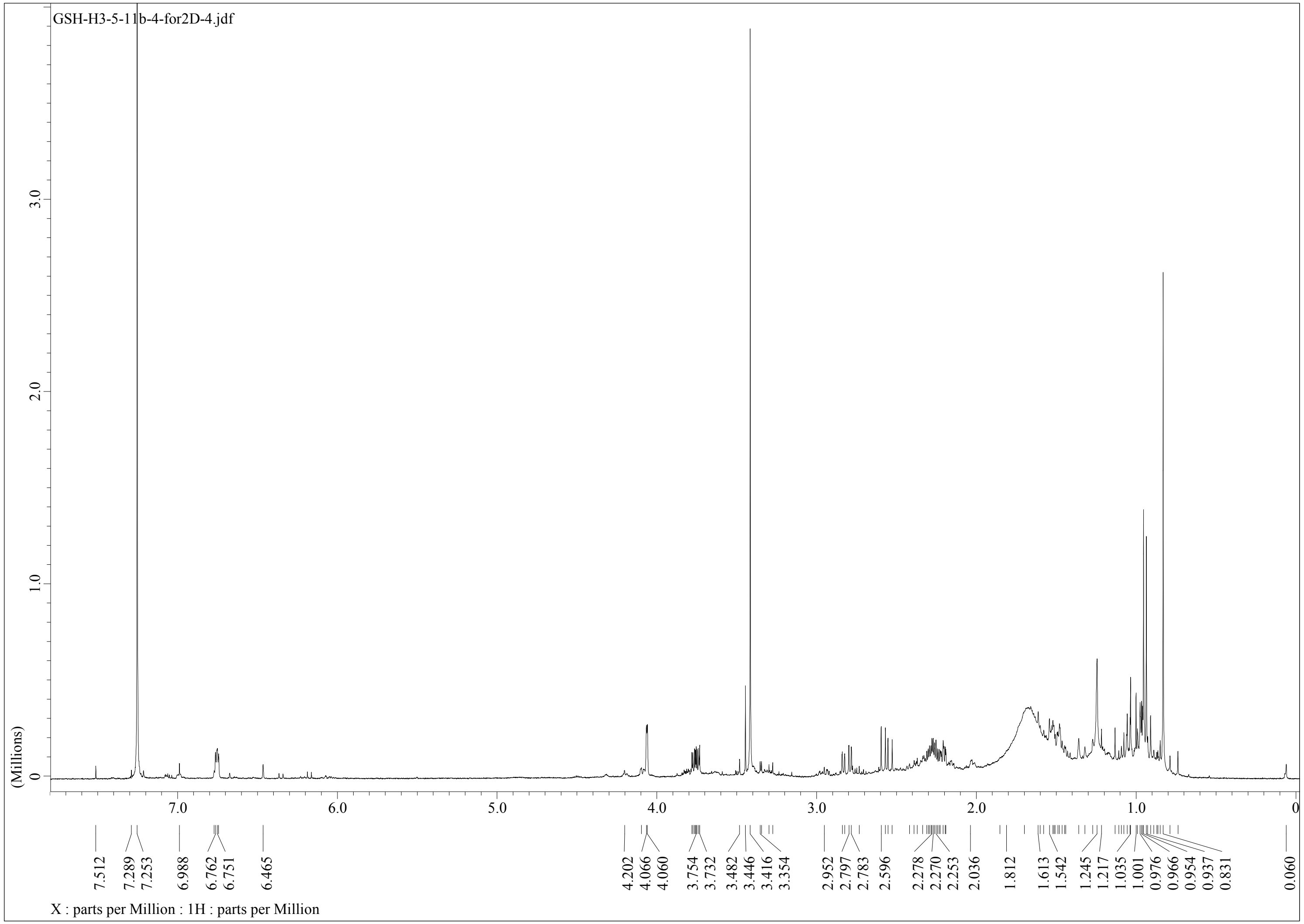

Figure S10. ^{1}H NMR (400 MHz, chloroform-*d*) spectrum of compound **3**.

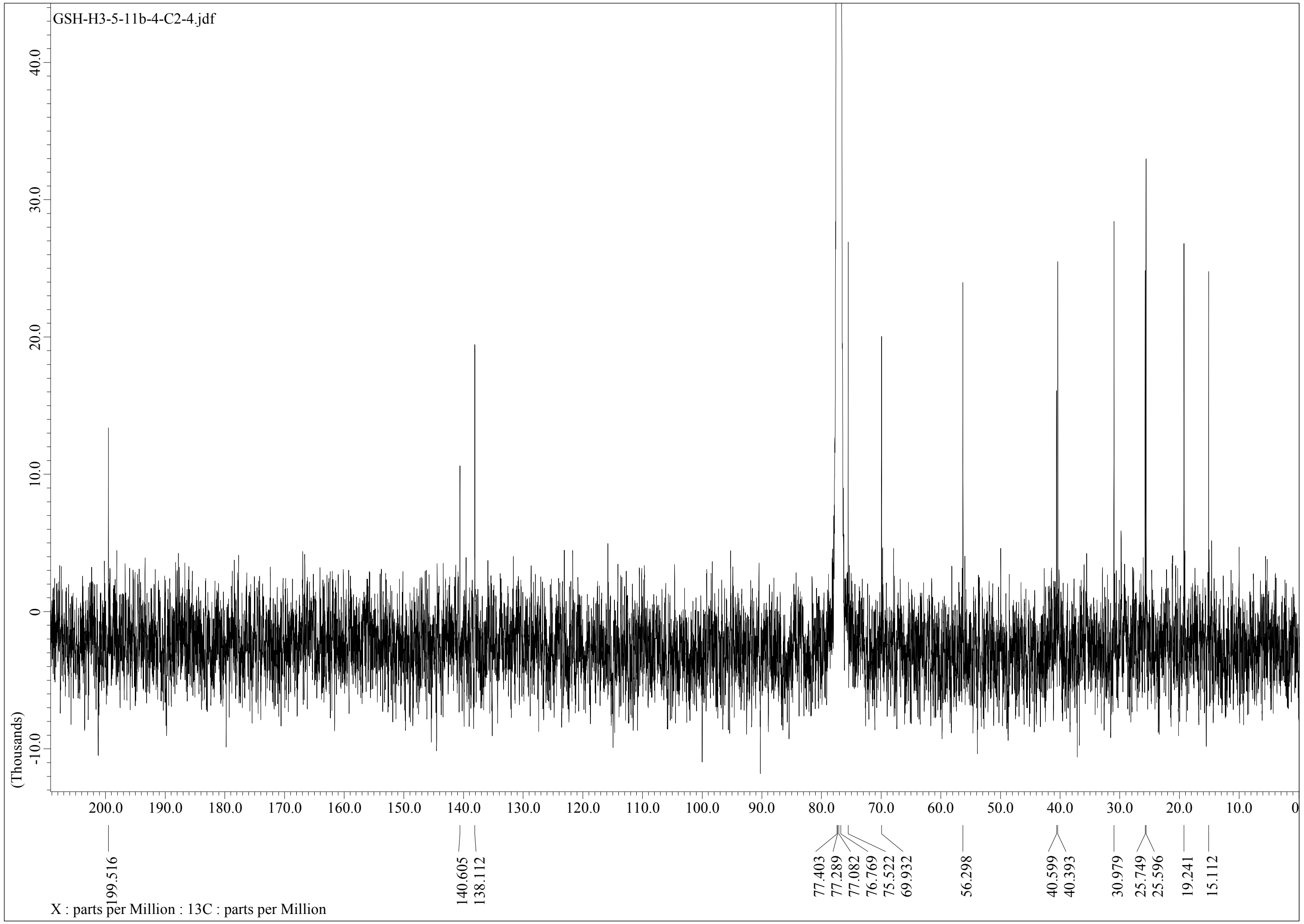

Figure S11. ^{13}C NMR (100 MHz, chloroform-*d*) spectrum of compound **3**.

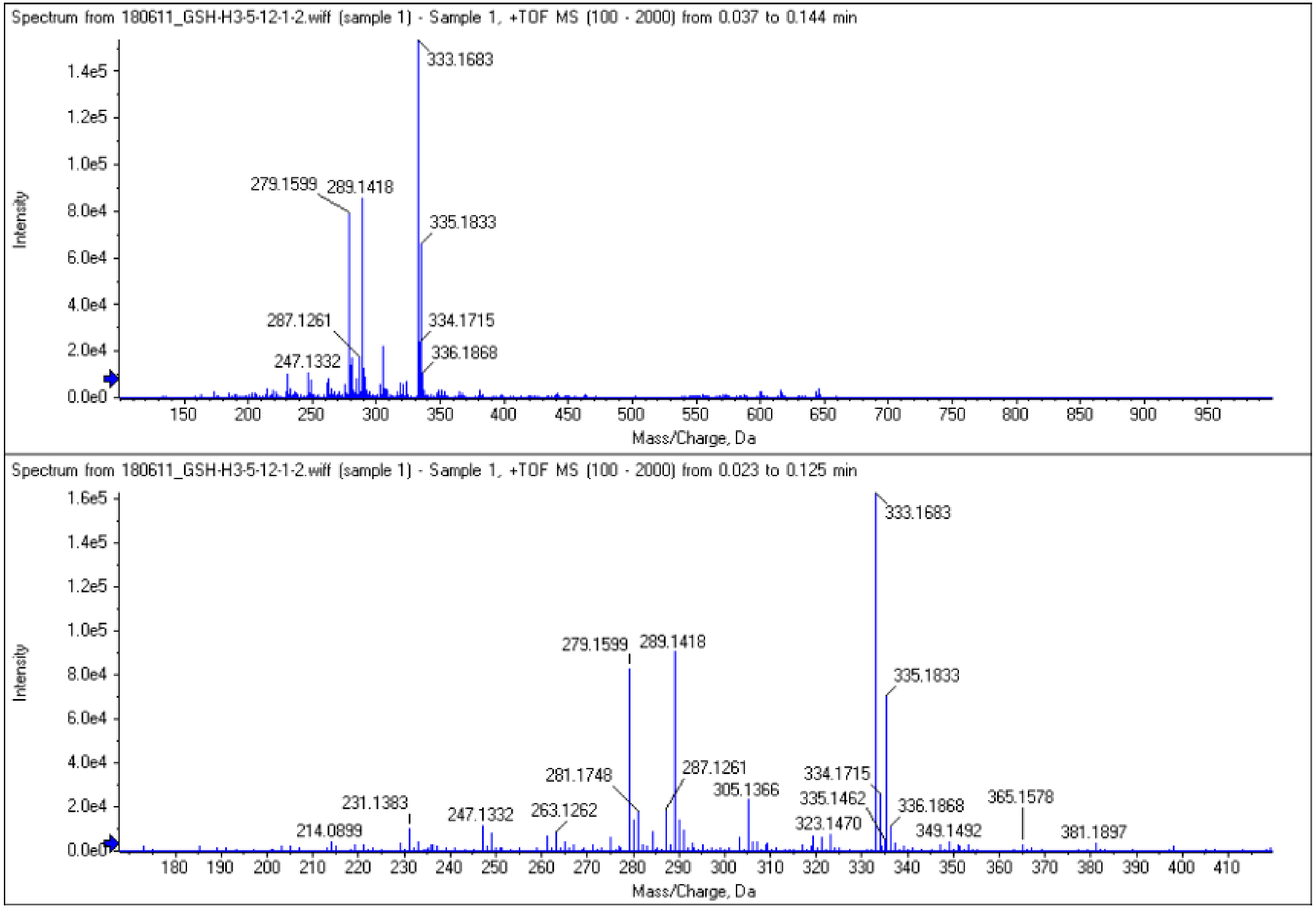

Figure S12. HRESIMS spectrum of compound **4**.

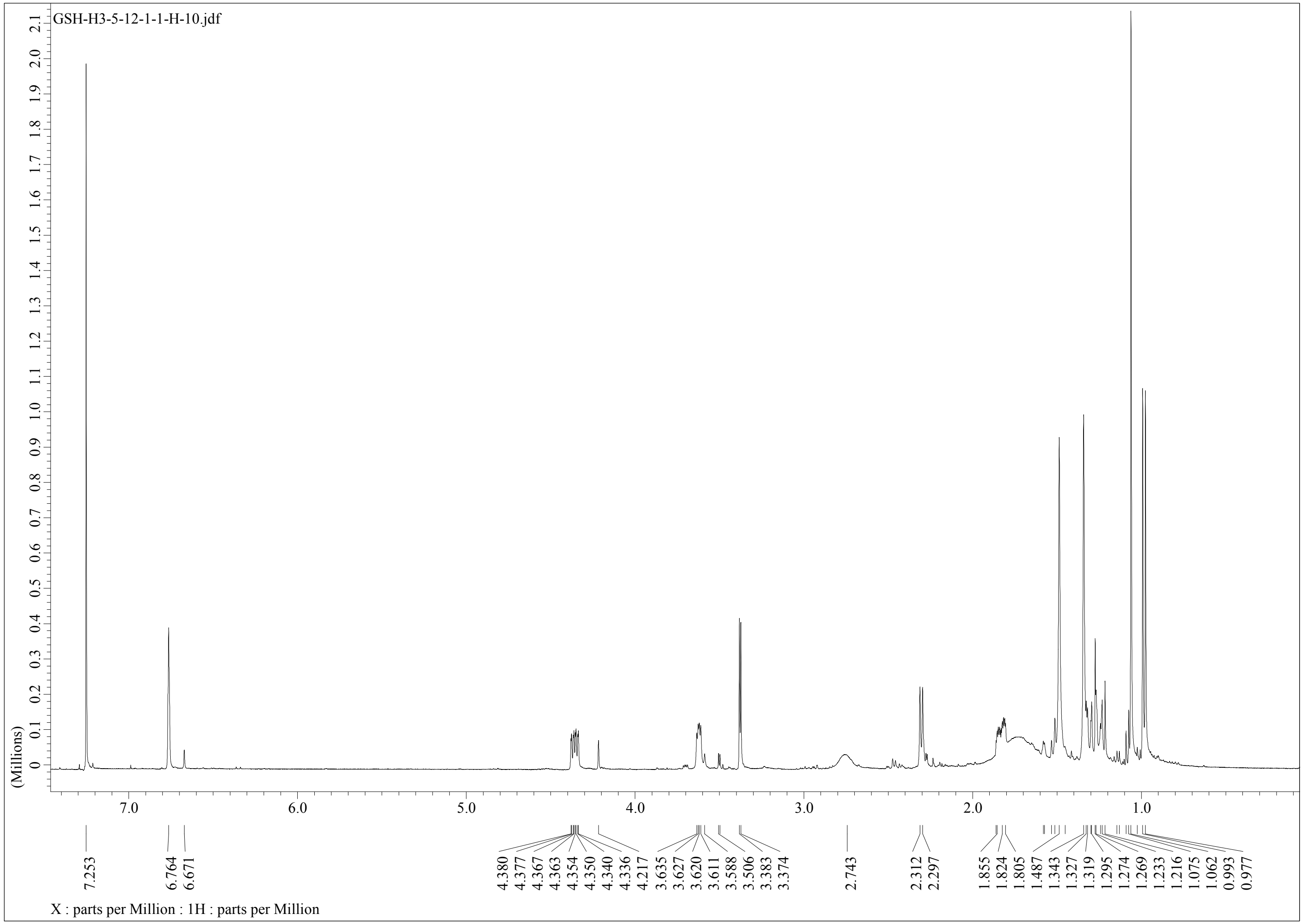

Figure S13. ^{1}H NMR (400 MHz, chloroform-*d*) spectrum of compound **4**.

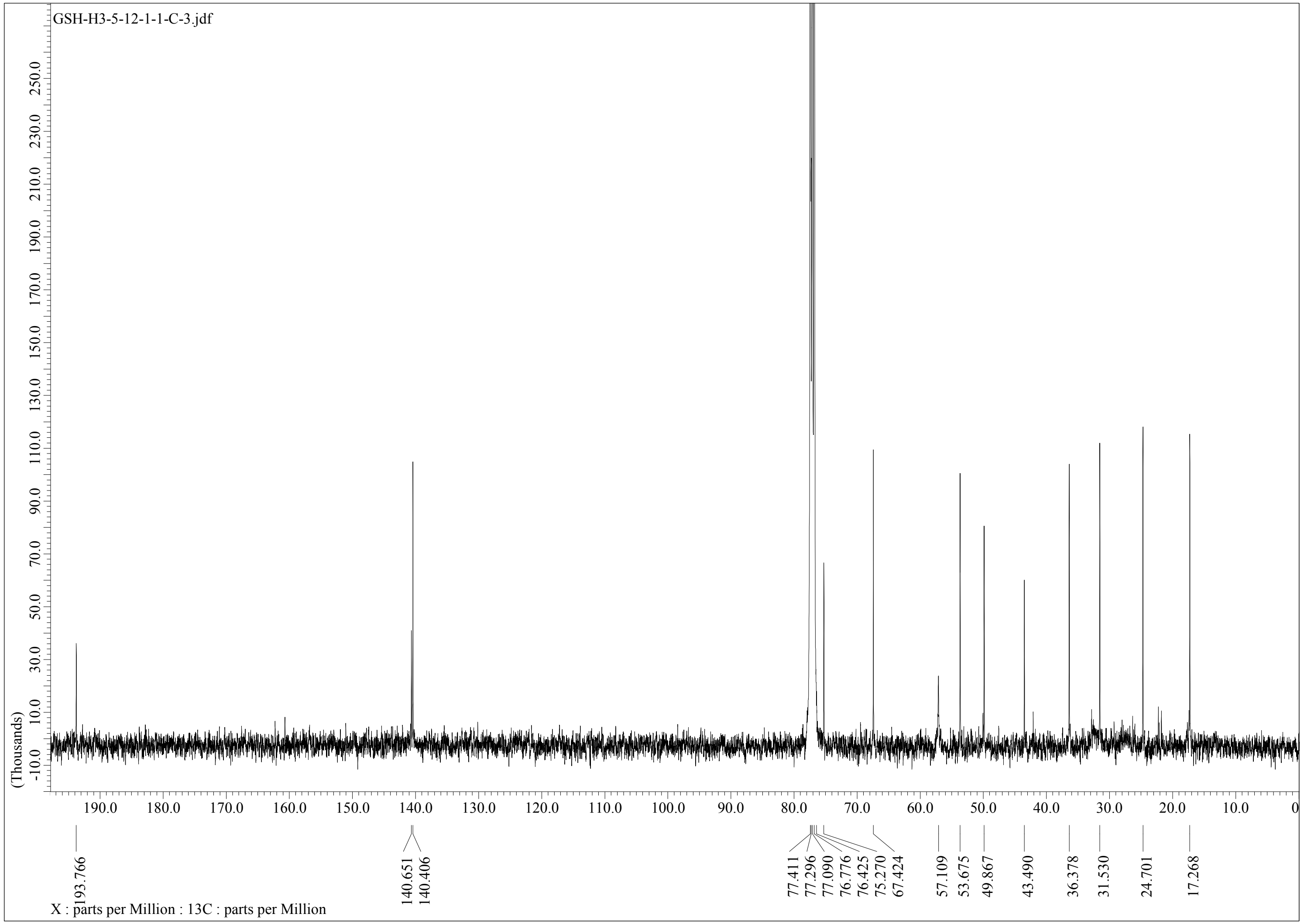

Figure S14. ^{13}C NMR (100 MHz, chloroform-*d*) spectrum of compound **4**.

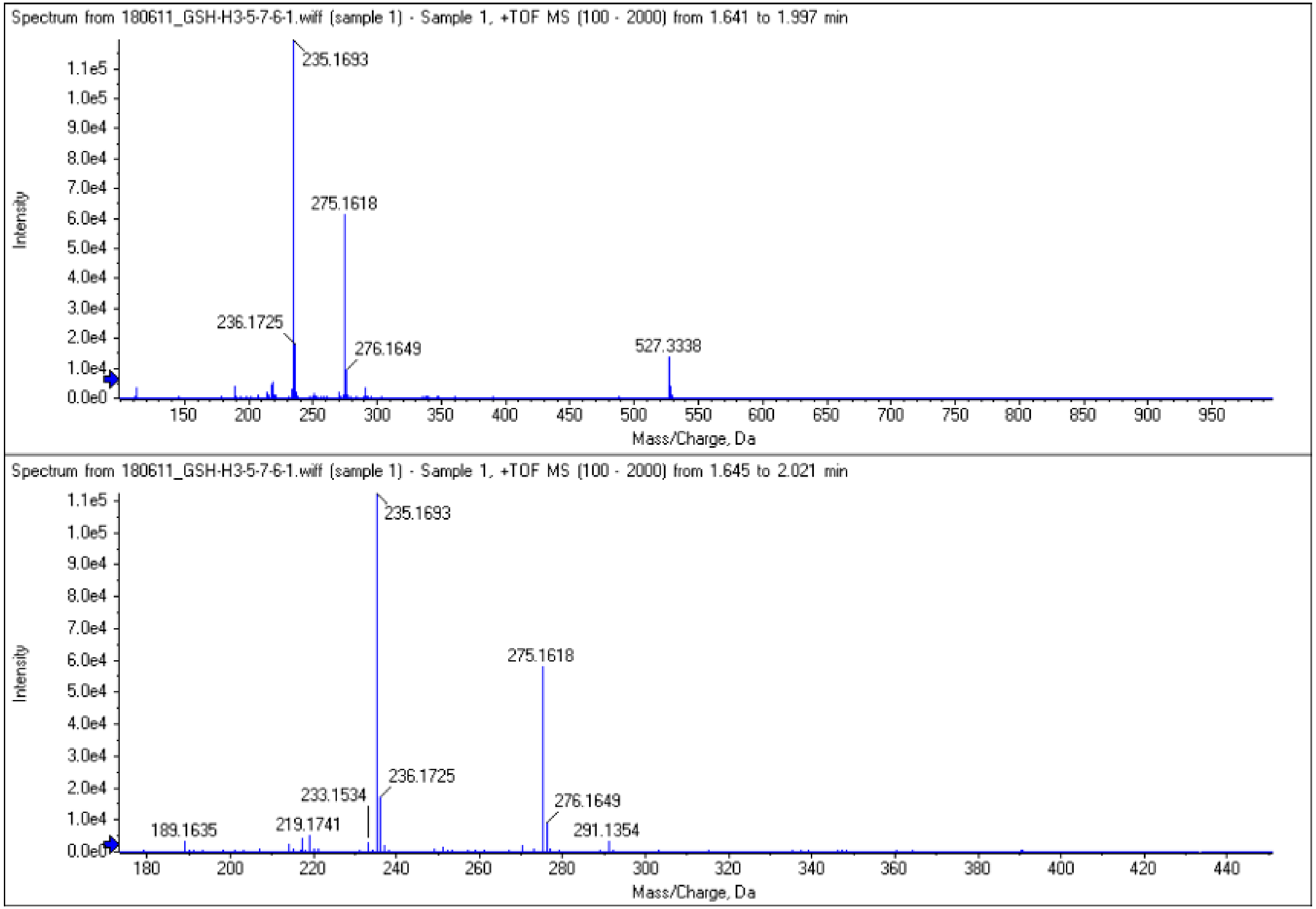

Figure S15. HRESIMS spectrum of compound **5**.

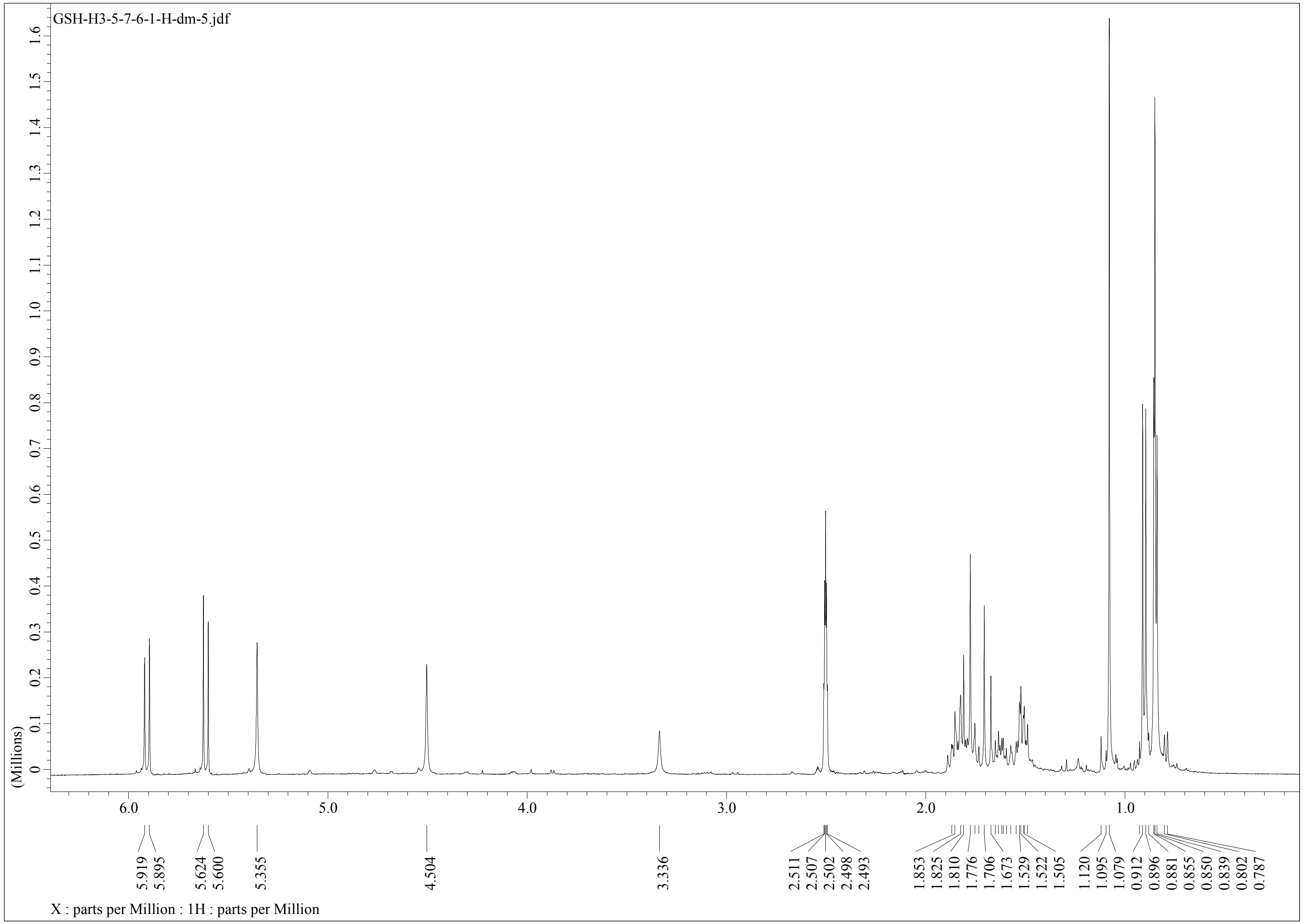

Figure S16. ^{1}H NMR (400 MHz, DMSO-d_6) spectrum of compound **5**.

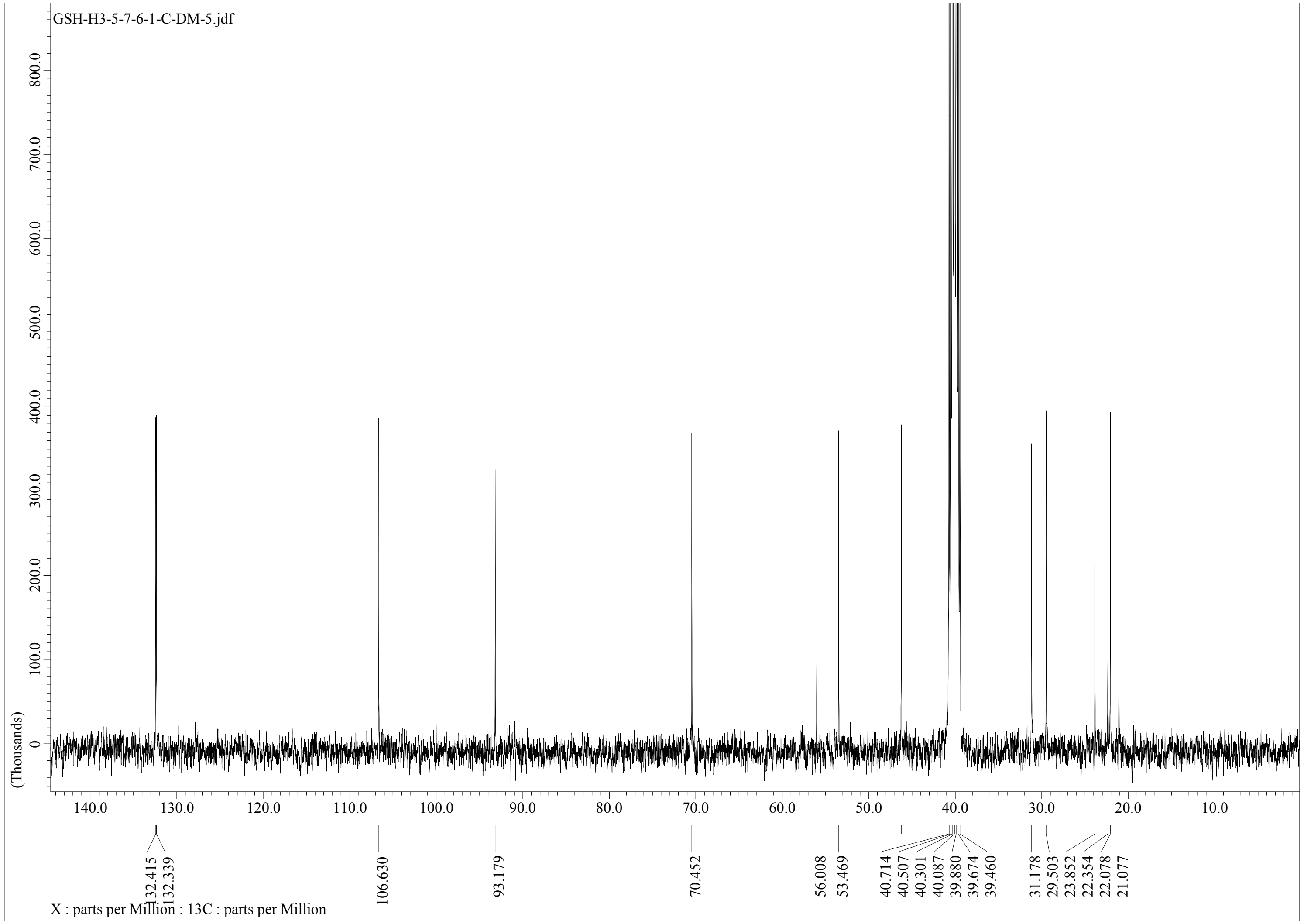

Figure S17. ^{13}C NMR (100 MHz, DMSO-d_6) spectrum of compound **5**.

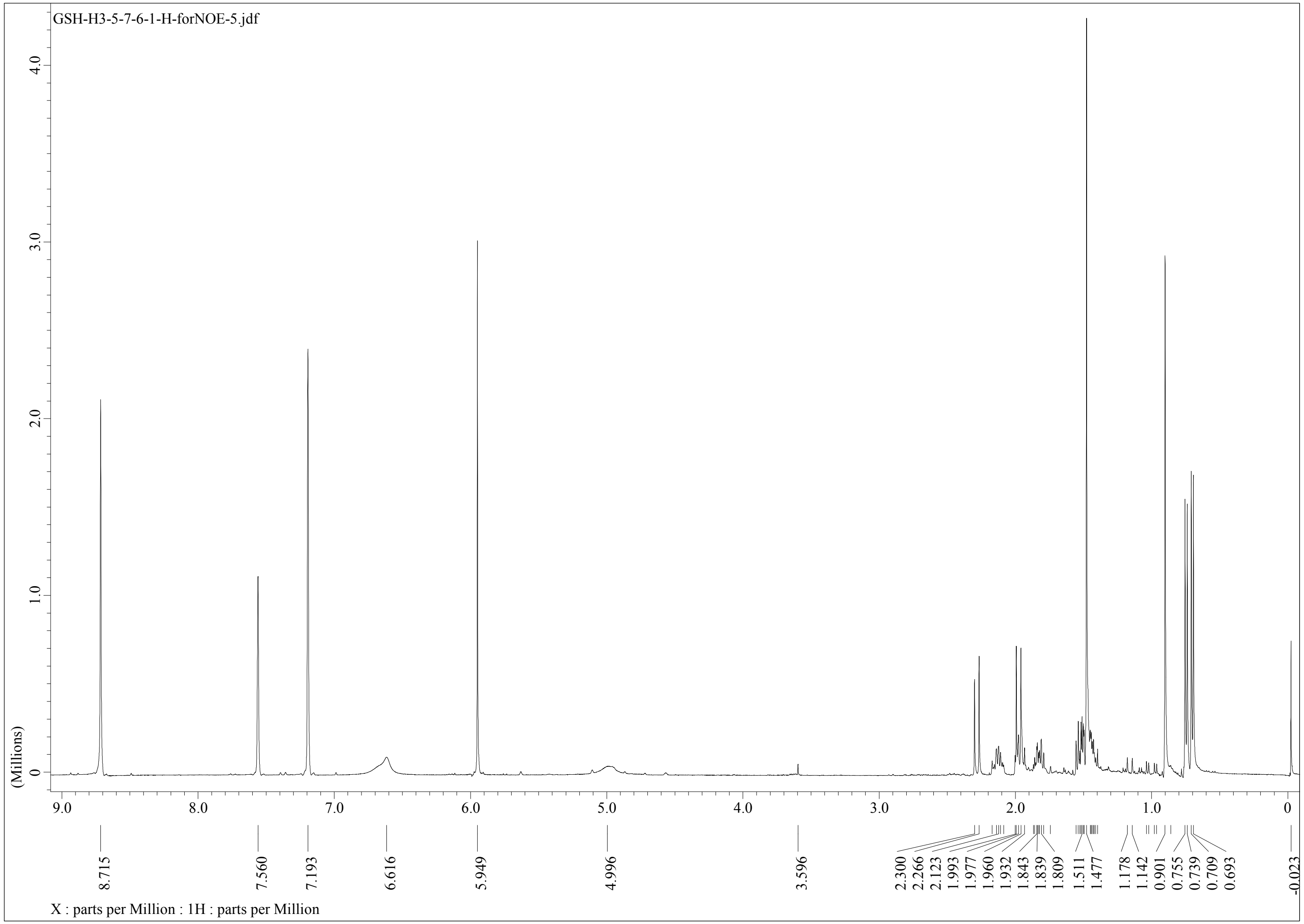

Figure S18. ^{1}H NMR (400 MHz, pyridine-d_5) spectrum of compound **5**.

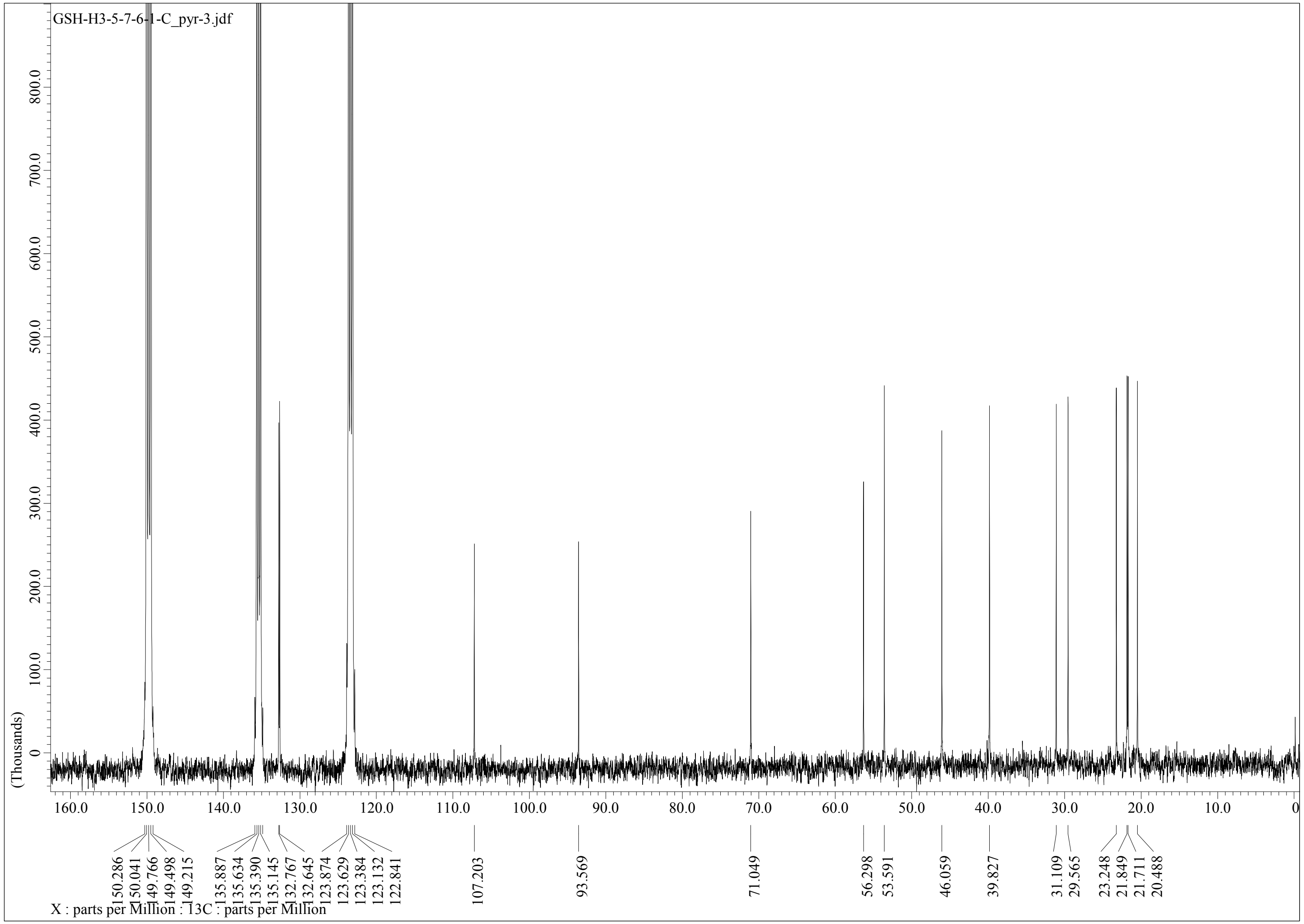

Figure S19. ^{13}C NMR (100 MHz, pyridine-d_5) spectrum of compound **5**.
